# Immune thrombocytopenia (ITP) World Impact Survey (I‐WISh): Impact of ITP on health‐related quality of life

**DOI:** 10.1002/ajh.26036

**Published:** 2020-12-19

**Authors:** Nichola Cooper, Alexandra Kruse, Caroline Kruse, Shirley Watson, Mervyn Morgan, Drew Provan, Waleed Ghanima, Donald M. Arnold, Yoshiaki Tomiyama, Cristina Santoro, Marc Michel, Serge Laborde, Barbara Lovrencic, Ming Hou, Tom Bailey, Gavin Taylor‐Stokes, Jens Haenig, James B. Bussel

**Affiliations:** ^1^ Department of Haematology Hammersmith Hospital, Imperial College London London UK; ^2^ Platelet Disorder Support Association Cleveland Ohio USA; ^3^ Patient representative for the UK ITP Forum Bolnhurst UK; ^4^ ITP Support Association Bolnhurst UK; ^5^ Academic Haematology Unit, Blizard Institute Barts and The School of Medicine and Dentistry London UK; ^6^ Department of Medicine Østfold Hospital Trust Kalnes Norway; ^7^ Department of Hematology, Institute of Clinical Medicine University of Oslo Oslo Norway; ^8^ Department of Medicine, McMaster Centre for Transfusion Research McMaster University Hamilton Ontario Canada; ^9^ Department of Blood Transfusion Osaka University Hospital Osaka Japan; ^10^ Hematology University Hospital Policlinico Umberto I Rome Italy; ^11^ Department of Internal Medicine, National Referral Center for Adult Immune Cytopenias Henri Mondor University Hospital, Assistance Publique Hôpitaux de Paris, Université Paris‐Est Créteil Créteil France; ^12^ OʼCyto Saint Loubes France; ^13^ Italian Association of Immune Thrombocytopenic Purpura Caprino Veronese Italy; ^14^ Department of Hematology Shandong University Jinan China; ^15^ Bespoke Team Adelphi Real World Macclesfield UK; ^16^ Novartis Pharma AG Basel Switzerland; ^17^ Division of Hematology/Oncology Weill Cornell Medicine New York New York USA

## Abstract

Immune thrombocytopenia (ITP) has a substantial, multifaceted impact on patients' health‐related quality of life (HRQoL). Data describing which aspects of ITP physicians and patients perceive as having the greatest impact are limited. The ITP World Impact Survey (I‐WISh) was a cross‐sectional survey, including 1507 patients and 472 physicians, to establish the impact of ITP on HRQoL and productivity from patient and physician perspectives. Patients reported that ITP reduced their energy levels (85% of patients), capacity to exercise (77%), and limited their ability to perform daily tasks (75%). Eighty percent of physicians reported that ITP symptoms reduced patient HRQoL, with 66% reporting ITP‐related fatigue substantially reduced patient HRQoL. Patients believed ITP had a substantial impact on emotional well‐being (49%) and 63% worried their condition would worsen. Because of ITP, 49% of patients had already reduced, or seriously considered reducing their working hours, and 29% had considered terminating their employment. Thirty‐six percent of patients employed at the time of the survey felt ITP decreased their work productivity, while 51% of patients with high/very high symptom burden reported that ITP affected their productivity. Note, I‐WISh demonstrated substantive impact of ITP on patients' HRQoL both directly for patients and from the viewpoint of their physicians. Patients reported reduced energy levels, expressed fears their condition might worsen, and those who worked experienced reduced productivity. Physicians should be aware not only of platelet counts and bleeding but also the multi‐dimensional impact of ITP on patients' lives as an integral component of disease management.

## 1 INTRODUCTION

Primary immune thrombocytopenia (ITP) is an autoimmune disorder characterized by reduced platelet counts (< 100 × 10^9^/L) and increased bleeding risk in the absence of another cause or disorder associated with thrombocytopenia.^1^ In adults, its prevalence ranges from 9.5 to 12.1 cases/100 000 in the United States, rising with increasing age.^2^, ^3^

The impact of ITP and its treatments on patient health‐related quality of life (HRQoL) may affect the entire spectrum of patients' lives, encompassing daily activities, emotional health, energy level, fatigue, and work productivity. The HRQoL is often reduced in patients with ITP compared with healthy controls.^4^, ^5^, ^6^ Compared with age‐matched and sex‐matched controls, patients with ITP have lower work productivity, more physician visits, and are more likely to take sick leave.^7^ These findings of reduced HRQoL in patients with ITP are largely undisputed. However, one patient survey reported only 11% of respondents felt ITP “often or extremely” affected their school or work activities, and only 7% reported non‐occupational activities were adversely affected by their disease.^8^ Another survey of patients with ITP and hematologists reported a reverse mismatched perception of disease impact on HRQoL;^9^ 71% of “ITP‐experienced” hematologists felt patient HRQoL was moderately to substantially impaired, whereas only 34% of patients reported any impairment.^9^ These findings may be consistent with a study of more than 400 patients showing that those with chronic ITP (disease duration ≥ 12 months) reported being less affected by ITP than patients with persistent disease (duration of ITP 3‐12 months). This suggests patients become so accustomed to a life with reduced HRQoL that they may think it is their “normal”.^1^, ^4^ This hypothesis has been validated in patients with ITP who rediscover their energy after treatment.^10^

There are limited data describing which aspects of ITP treating physicians and patients perceive as having the greatest impact on HRQoL nor is there a clear, overall description of the full range of effects on patients.

This report presents the results of the international, online, ITP World Impact Survey (I‐WISh) completed by 1507 patients and 472 physicians, summarizing the impact of ITP on various aspects or dimensions of patients' HRQoL, and comparing patient and physician perceptions of symptoms and disease management. Survey data reporting on the patient journey and symptoms associated with ITP will be reported in a separate manuscript (Cooper et al., manuscript in preparation).

This report focusses on functional and psychological/emotional impact of ITP on quality of life, work and activities, satisfaction with treatment, and the relationship between the patient and physician.

## 2 METHODS

### 2.1 Study design

Thus, I‐WISh was a cross‐sectional survey of patients with ITP and hematologists or hemato‐oncologists, who treat patients with ITP, from 13 countries (Canada, China, Colombia, Egypt, France, Germany, India, Italy, Japan, Spain, Turkey, UK, and US). Patients ≥ 18 years old, diagnosed with ITP, who had not previously completed an I‐WISh survey,^11^ were invited to participate.

Patient and physician surveys were developed by a steering group that included hematologists clinically expert in ITP, and patient advocates representing several different countries' ITP patient associations. The patient questionnaire comprised six sections and collected information on demographics and diagnosis (seven questions), symptoms of ITP (four questions), HRQoL and emotion associated with ITP (12 questions), impact of ITP on work, finances, and support (15 questions), treatment received (17 questions), and patient and physician relationship (seven questions). The physician survey was similar in many aspects and comprised six sections that collected information on demographics (two questions), diagnosis of ITP and patient caseload (seven questions), symptoms of ITP (five questions), impact of ITP on aspects of patients' physical, emotional, HRQoL, and social health (11 questions), treatment patterns (13 questions), and patient and physician relationship (four questions). There were two questions about the emotional impact of ITP and the responses were based on a 7‐point Likert scale (1 = never, 7 = a great deal). There was one additional question on the need for professional support.

Patients also completed the newly‐developed ITP Life Quality Index (ILQI)^12^ that included 10 questions on impact of ITP on: work or studies, time taken off work or education, ability to concentrate, social life, sex life, energy levels, ability to undertake daily tasks, ability to provide support, hobbies, and capacity to exercise. The response options were “never”, “sometimes”, “more than half the time”, and “all of the time”.

Patient surveys were sent by mass email to patient support networks and physicians who were requested to disseminate the survey to patients. Physician surveys were emailed from local fieldwork agencies. Surveys took approximately 30 minutes to complete. Fully de‐identified respondent information was collated and aggregated by local fieldwork partners such that surveys were unlinked and anonymized. The surveys and details of the survey methods, including how patients and physicians were identified, have been outlined in supplementary material.

Survey materials and the study protocol were reviewed and approved by central Institutional Review Boards (IRB) in both Europe and North America. Patients and physicians were given an overview of the study and the ethical approval details; those who wished to participate had to provide consent via a tick/check box before initiating.

### 2.2 Statistical analyses

Patient and physician survey data were analyzed separately using descriptive statistics. No formal hypotheses were tested. The objective of this research was to conduct a global study with a sample size of over 1500 patients to understand the burden of living with ITP. As finding a representative, well‐matched control group would have been very difficult given the nature of the study, we opted to evaluate as many patients as possible in this exploratory analysis. This also enabled us to meaningfully explore various subgroups.

Subgroups that were explored included sex (males vs females), age (18‐49 vs ≥ 50 years), and symptom burden (low, moderate, high, and very high). Symptom burden score for the time of ITP diagnosis was retrospectively estimated by the patient when they completed the survey. The overall symptom burden was calculated by summarizing individual symptom severity scores and then dividing into quartiles where Q1 = lowest symptom burden and Q4 = highest symptom burden (supplementary methods).

## 3 RESULTS

The I‐WISh survey was completed by 472 physicians and 1507 patients between December 2017 and August 2018. The largest number of patients and physicians were recruited from the US, China, and the UK (Table S1). Physicians had a mean (SD) ITP patient caseload of 34 (50) and saw a mean (SD) of 18 (36) newly diagnosed patients in the past year (Table S2).

The mean age of patients with ITP was 47 years, 65% were female, and respondents had been diagnosed with ITP a median of 5 years earlier (Table S2). Forty‐eight percent of patients reported a high or very high symptom burden at diagnosis (Table S2).

### 3.1 Impact of ITP on functional aspects of patients' HRQoL

**FIGURE 1:**
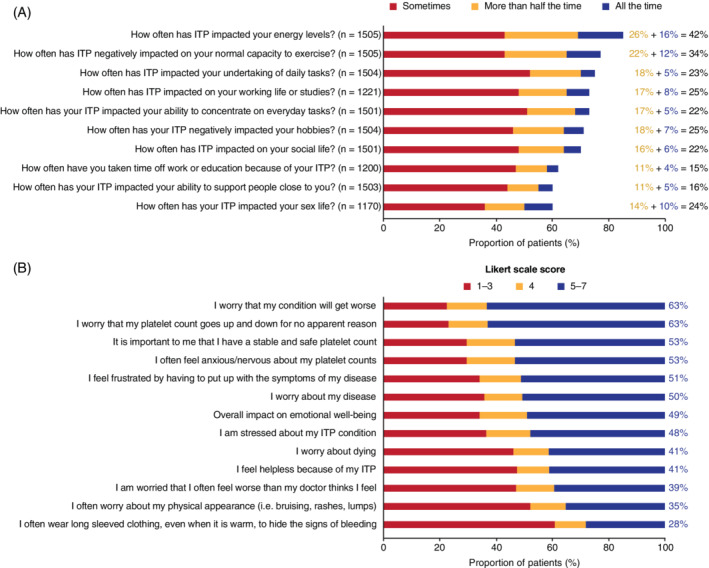
A, Proportion of patients reporting an impact of ITP on daily living (ILQI). ITP, immune thrombocytopenia. n = number of respondents. Note: The remaining respondents all replied “never”. B, Patient‐perceived factors driving a psychological/emotional impact in ITP. ITP, immune thrombocytopenia. N = 1507 for all questions. There were “not stated” responses for two questions: “It is important to me that I have a stable and safe platelet count” (n = 2) and “I worry that my platelet count goes up and down for no apparent reason” (n = 1). Note: % values refer to the proportion of patients scoring 5‐7 on a 7‐point Likert scale indicating the greatest psychological/emotional impact [Color figure can be viewed at wileyonlinelibrary.com]

Within the I‐WISh survey, the ILQI asked 10 questions that explored the impact of ITP on aspects of patients' daily lives within the last month (Figure 1A). Sixty percent, or more, of patients answered “sometimes” to “all the time” to all 10 questions. The aspects most affected (“more than half the time” or “all the time”) were energy levels (42%; n = 631/1505) and ability to exercise (34%; n = 515/1505), with the other eight aspects being most affected between 15% and 25% of the time. Fewer patients recruited by physicians (35%) reported that ITP affected their energy levels compared with patients recruited by PAGs (47%). Seventy percent of patients reported some impact of ITP on their social lives and 60% reported impact on their sex lives. More than one‐third of patients had high or very high symptom burden at diagnosis (n = 590, 39%) and 97% (n = 572) of these patients stated that ITP reduced their energy levels at some time.

Similar to the patient response, where 75% of patients felt the ability to undertake daily tasks was impacted by their ITP at least sometimes, two‐thirds (63%, n = 297/468) of physicians felt that ITP had a negative effect on the overall level of physical activity in their patients (a score ≥ 5 on a 7‐point Likert scale; 7 = a great deal).

Fifty percent of patients reported fatigue as a symptom at the time of survey completion and 73% reported difficulty concentrating at least sometimes. Physicians (n = 465) reported that 38% of their patients felt fatigued and, of these, considered that 46% experienced a high level of fatigue (score ≥ 5 on a Likert scale 1‐7; 7 = completely fatigued). Eighty percent (n = 378/472) of physicians reported that ITP symptoms reduced patient HRQoL, and 66% (n = 313/472) reported that ITP‐related fatigue substantially reduced patient HRQoL (score ≥ 5 on a Likert scale 1‐7; 7 = a great deal).

### 3.2 Impact of ITP on patients' psychological and emotional well‐being

Almost half of patients (49%, n = 739/1507) felt that ITP negatively impacted their psychological and emotional well‐being (score ≥ 5 on a Likert scale 1‐7; 7 = a great deal). The issues most affected were concerns that their condition would worsen (63%; n = 954/1507), about unexplained fluctuations in platelet levels (63%; n = 949/1507), the importance of having stable and safe platelet levels (53%; n = 803/1507), and feeling anxious/nervous about their platelet counts (53%, n = 803/1507) (Figure 1B). Fifty‐one percent of women and 46% of men, and 53% of younger patients (aged 18‐49 years) and 44% aged ≥ 50 years stated that ITP negatively impacted their emotional well‐being.

Seventy percent (n = 411/590) of those with high/very high symptom burden at survey completion reported a negative impact on their emotional well‐being, with the main issues being the same as those for all ITP patients: worries that their condition will worsen (80%; n = 473/590), concerns about unexplained fluctuations in platelet levels (76%; n = 446/590), and frustration about having to tolerate their symptoms (72%; n = 425/590). Similar to patient responses, 69% of physicians (n = 316/459) felt that patients' anxiety about their platelet levels had a substantial negative impact on their emotional well‐being (score 6 or 7 on a Likert scale 1‐7; 7 = a great deal). Thirty‐nine percent of patients believed their doctor thought they felt better than they actually did.

Forty‐one percent of patients (n = 618/1507) were worried about dying (score ≥ 5 on a Likert scale 1‐7; 7 = a great deal); this proportion was the same for both women and men. Forty‐four percent of younger patients (aged 18‐49 years) reported worries about dying compared with 38% of older patients (aged ≥ 50 years). Forty‐eight percent (n = 220/459) of physicians felt that their patients were worried about dying from ITP.

There was evidence of concern about physical appearance, however patients did not rate this concern as high as other issues. Thirty‐five percent (n = 527/1507) of patients indicated substantial concern about physical appearance (ie, bruising, rashes, and lumps), and 28% (n = 422/1507) of patients reported wearing long‐sleeved clothing to hide evidence of bleeding (score ≥ 5 on a Likert scale 1‐7; 7 = a great deal) (Figure 1B). When separated by gender, 31% of males were not concerned about physical appearance (Likert score of 1) compared with 22% of females; however, the proportions of women and men reporting concern about their physical appearance were similar (score ≥ 5 on a Likert scale 1‐7; 7 = a great deal). Thirty‐nine percent of younger patients (aged 18‐49 years) voiced concerns about their physical appearance compared with 30% of older patients (aged ≥ 50 years).

### 3.3 Impact of ITP on work and activities

The Organisation for Economic Co‐operation and Development (OECD) indicates that, across its 37 member countries, 68.9% of the population of working age (15 to 64 years of age) are employed and that 16.5% of this total are in part‐time employment.^13^ At survey completion, 60% of respondents reported that they were employed; 44% (n = 661/1507) were in full‐time employment and 16% (n = 235/1507) in part‐time employment (Table 1).

**TABLE 1:** Current employment status

Current employment status, n (%) (n = 1507)	Patients N = 1507
Working full‐time	661 (44)
Working part‐time	235 (16)
Retired or not working and not seeking employment	344 (23)
Homemaker	102 (7)
Student	68 (5)
Long‐term sick leave or disability	48 (3)
Not working, seeking employment	37 (2)
Other	12 (1)

**FIGURE 2:**
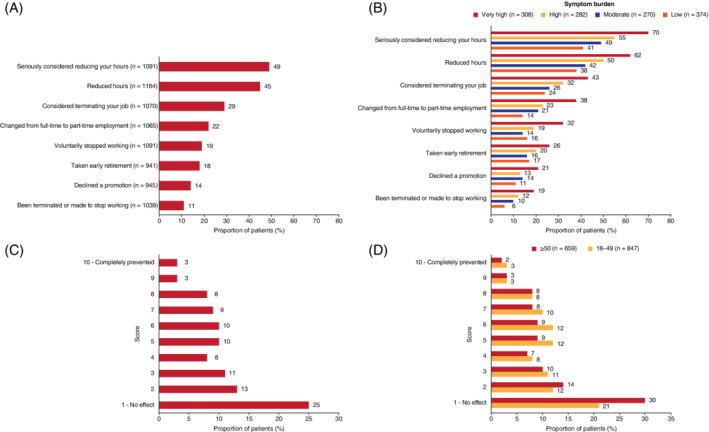
A, Effect of ITP on employment status. B, Effect of ITP on employment status by symptom burden. ITP, immune thrombocytopenia. C, Effect of ITP on regular activities. D, Effect of ITP on regular activities by age. ITP, immune thrombocytopenia [Color figure can be viewed at wileyonlinelibrary.com]

Forty‐nine percent of respondents (n = 538/1091) reported that they had reduced or seriously considered reducing their working hours because of their ITP, with 29% (n = 307/1070) reporting that they had considered terminating their employment (Figure 2A). Overall, 11% of patients were forced to stop working because of their ITP, with this figure rising to 19% for patients with a very high symptom burden. Fourteen percent of respondents had declined a promotion because of their ITP, increasing to 21% for patients with a very high symptom burden (Figures 2A,B).

Over one‐third of patients who were employed at the time of the survey (36%; n = 319/896) felt ITP negatively affected on their productivity at work (score ≥ 5 on a scale of 1‐10; 10 = completely prevented from working), especially those with high or very high symptom burden. Proportions of women and men reporting an impact of ITP on their work productivity were similar, and 40% of younger patients (18‐49 years; median duration of ITP 4 years) reported a high impact on productivity at work compared with 27% of patients aged ≥ 50 years (median duration of ITP 7 years).

Forty‐three percent of all patients reported that their ITP affected regular activities outside of work (described as work around the house, shopping, childcare, exercise, and studying [score ≥ 5 on a scale of 1‐10; 10 = completely prevented productivity]) (Figure 2C). Proportions of women and men who reported an impact of ITP on their regular activities were similar. Forty‐eight percent of younger patients (18‐49 years) reported an impact on regular activities compared with 39% of patients aged ≥ 50 years (Figure 2D). Fifty‐two percent (n = 239/461) of physicians felt that ITP had a negative impact on their patients' ability to undertake daily activities (food preparation, housework, gardening, childcare, and oral hygiene).

### 3.4 Patient satisfaction and perception of treatment

Patients were asked about their satisfaction with the current pharmacological interventions being used for treating their ITP. Patients who were being treated with anti‐CD20 agents reported the greatest overall satisfaction regarding control of their ITP (79%; n = 55/70), with similar results for thrombopoietin receptor agonists (76%; n = 182/240), intravenous immunoglobulin (69%; n = 77/112), but less positive results for corticosteroids (53%; n = 194/368). Only 38% (n = 95/250) of patients who had undergone splenectomy reported being satisfied with the procedure (score ≥ 5 on a Likert scale 1‐7; 7 = strongly agree). Nine percent (n = 10/111) of patients with normal platelet counts after splenectomy reported that they regretted having the operation even though it worked (score ≥ 5 on a Likert scale 1‐7; 7 = strongly agree), with 14% (n = 16/111) saying it was causing them other problems.

Patients (n = 1497‐1507) and physicians (n = 472) both identified the ability to offer sustained remission or cure of ITP (patient score 90/100; physician score 82/100; 100 = highest importance) and reduction of bleeding risk (patient score 87/100; physician score 86/100) as the attributes with the highest importance when making decisions about treatment and management. This perception was similar by sex or age group. Keeping side effects to a minimum and avoiding immunosuppression were also very important. Patients and physicians were very well aligned in their perceptions of what was most important.

### 3.5 Treatment goals and relationship with physician

**FIGURE 3:**
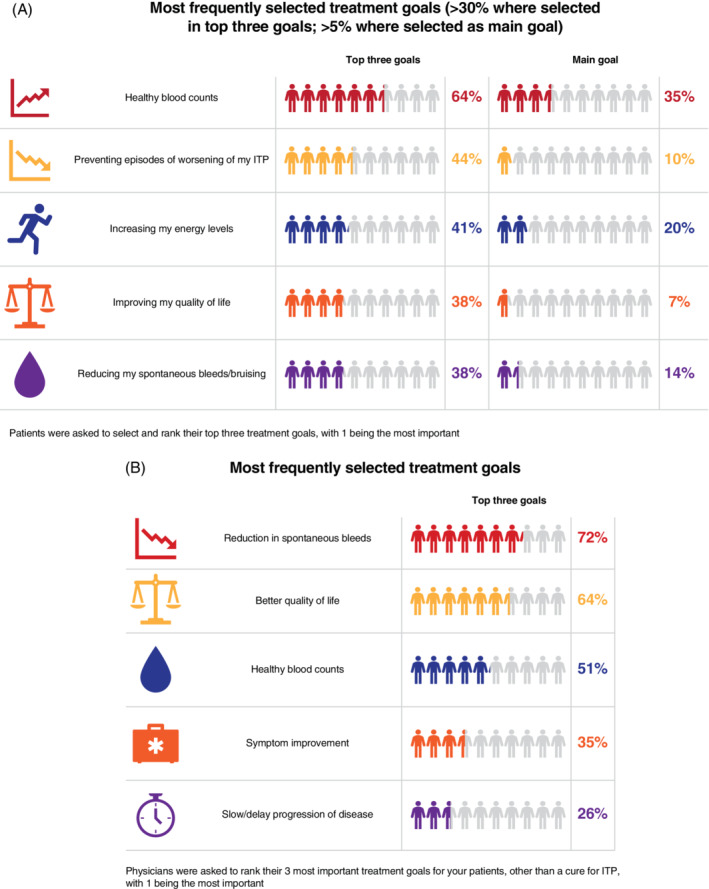
Top three treatment goals identified by A, patients and B, physicians. Most frequently selected treatment goals (patients and physicians were asked to select and rank their top three treatment goals) [Color figure can be viewed at wileyonlinelibrary.com]

Outside of sustained remission or a cure for ITP and reducing side effects, the three most important treatment goals identified by patients were “healthy blood counts” (64%; n = 968/1507), “preventing episodes of worsening of my ITP” (44%; n = 661/1507), and “increasing my energy levels” (41%; n = 613/1507) (Figure 3A).

For physicians, the top three goals (beyond a cure for ITP) when treating patients with ITP were “reduction of spontaneous bleeding” (72%; n = 340/472), “better quality of life” (64%; n = 300/472), and “healthy blood counts” (51%; n = 240/472) (Figure 3B).

Eighty‐nine percent (n = 422/472) of physicians felt that patients were satisfied with the treatment goals that had been set, and 81% (n = 382/472) felt that their patients were satisfied with the ease and convenience of prescribed medication. Seventy‐four percent (n = 1118/1505) of patients felt their physician was aware of their treatment goals, and 70% (n = 1058/1506) somewhat/strongly agreed that their current treatment was helping them to achieve their goals; the proportions by gender (67% women and 76% men) and age groups were similar. Seventy‐six percent (n = 1148/1507) of patients reported they were satisfied that the physician responsible for their care understood and supported their treatment goals, and 77% (n = 1157/1506) reported they were satisfied that their physician considered their needs when planning their treatment goals. Satisfaction with support, and consideration of needs was similar between sex and age groups.

Physicians felt that their patients had a high level of satisfaction regarding aspects of their care (score ≥ 5 on a Likert scale 1‐7; 7 = completely satisfied); 86% (n = 407/472) and 87% (n = 410/472) of physicians felt that their patients were satisfied with their treatment decisions and management of their condition, respectively. Seventy‐eight percent (n = 1180/1506) of patients reported satisfaction with the management and treatment of their ITP (score ≥ 5 on a Likert scale 1‐7; 7 = completely satisfied). Physicians felt patients were generally satisfied (88%; n = 417/472) with the level of communication about their disease and treatment, while 79% (n = 1198/1507) of patients reported that they were satisfied with the level of communication from their physician about their disease and treatment. Satisfaction with management and treatment of ITP and physician communication about disease and treatment were similar for sex and age group.

## 4 DISCUSSION

In this online survey, patients and physicians reported considerable impact of ITP on patient HRQoL, with reduced energy levels and capacity to exercise, and limited ability to perform daily tasks. Patients also described substantial influence on their emotional well‐being and many worried their condition would worsen. Those patients who were working felt ITP negatively affected their productivity, with nearly half having already reduced, or seriously considered reducing, their working hours. Thirty‐six percent of patients employed at the time of the survey felt ITP decreased their productivity at work.

Reduced QoL was first described in adults with ITP in 2008 and this has been confirmed many times thereafter.^4^, ^5^, ^6^ Generic tools like the SF‐36 have indicated a reduced QoL in patients compared with controls or the general population, in particular for physical and social activities.^4^, ^5^, ^6^, ^14^ These validated tools are useful in exploring overall QoL. However, they are not disease‐specific and do not identify the key factors that have the greatest impact on the HRQoL in patients with ITP. The 10‐scale ITP‐patient assessment questionnaire (PAQ), which was not available at the time of design of this study, suggested that successful treatment of ITP was associated with improvement in HRQoL.^15^

The I‐WISh survey, which included the 10‐question ILQI, is a novel, very extensive QoL questionnaire which identifies the degree of impact that ITP has on patients' HRQoL, emotional health, social, personal, and work life. Concerns about unstable platelet counts, low energy levels, inability to exercise, and reduced participation in hobbies and work had the greatest negative impact. While most patients reported “good health”, nonetheless half of patients reported a negative impact on their emotional well‐being that worsened with increasing burden of disease and was often substantial. This “overly good” report about health may reflect that they were not bleeding at the time of survey completion, but probably more so that they were acclimated to their reduced state of energy, and did not consider this in their “health” assessment, perhaps not realizing it was at least partially connected with the ITP. People also have the tendency to say that they are “fine” when asked.

Previous evidence reported fatigue in 1/5 to 1/2 of adults with ITP.^9^, ^16^ Note, I‐WISh found that 50% of patients reported fatigue at the time of survey completion, and physicians believed that 38% of their patients experienced fatigue. A control sample population similar to those with ITP is unfortunately not available for comparison of levels of fatigue in the general population.

Previous evidence from a discussion group of 23 patients found that patients reported restrictions in their social activities due to bruising.^15^ So, I‐WISh also identified concerns about physical appearance, including bruising, as an issue in 35% of patients with ITP to the extent of wearing long‐sleeve clothing. Health professionals working with patients with ITP need to consider the impact of physical appearance and fatigue on HRQoL, and adjust their management accordingly. Surprisingly, this was not different between males and females. As initially reported using the ITP‐PAQ,^15^ ITP has an impact on sex life as well as social life and this was confirmed here in 60% and 70% of patients, respectively.

Work and productivity were negatively impacted by ITP; almost half of patients reported they had reduced, or seriously considered reducing, their working hours because of ITP. Work hours were especially impacted for patients with a very high symptom burden, with over two‐thirds of these patients seriously considering reducing their work hours, and 62% actually reducing their hours at work at some point. Up to 25% declined a promotion, had to take early retirement, or quit their job. This can create conflict in having to work to avoid long‐term financial burden on the patient and their family. There is also the conflict of having to take time off to visit (and pay) the physician. This area requires further exploration, but ITP clearly had quite a substantial effect in the approximately 20% of patients with a very high symptom burden.

Differences between physician and patient viewpoints on HRQoL have been reported. This survey reflected the increased awareness of reduced HRQoL and low energy in patients with ITP by physicians who were experienced in the care of ITP. Given how many ITP patients these physicians see per year, more than 40 on average, it is not surprising that 80% of physicians reported that they felt ITP symptoms reduced their patientsʼ QoL. In general, the differences in their impressions and that of their patients was, remarkably, relatively small and well‐aligned. The leading hypothesis to explain the absence of greater complaints by patients is that patients may be so acclimated to their condition (median 5 years of ITP) that they fail to realize that their energy level and QoL are reduced compared to “normal” because their normal has been low for so long.^4^ This suggests that it is not enough to accept a patientʼs self‐report that they feel well and necessitates an exploration into their activities, for example using the ILQI. Reduced HRQoL may be a reason to consider both greater exploration of the underlying reason and treatment of the platelet count in a non‐bleeding patient.

Eighty‐five percent of physicians felt their patients experienced anxiety about platelet counts, whereas this was identified as an issue in 53% of patients. Nonetheless, unexplained fluctuations in platelet counts were identified as a major patient concern. Similar proportions of physicians and patients identified fear of dying from ITP as an issue (48% vs 41% respectively). It is the first time that concerns about dying from their disease have been studied as a specific issue for patients with ITP. Given the very low rate of death in adequately managed, non‐elderly patients with ITP, this fear is disproportionate to the factual risk. This may be a result of the physician having to explain at diagnosis the risk of fatal bleeding to the patient and his/her family and this being etched into their consciousness and reinforced whenever (if ever) the platelet count falls. The origin of this concern may also feasibly be the internet.

Although most patients (64%) reported a high score for their current health state (score 5 to 7 on a 7‐point Likert scale; 7 = excellent health), with only 15% reporting a low score (1‐3), the evidence suggests that they still frequently have important complaints.

So, I‐WISh was a large international survey of 1507 patients with ITP and 472 physicians from 13 countries. Selection bias is a limitation of this study because patients completing the survey were likely to be motivated and potentially have more severe disease than patients who chose not to complete the survey. Recall bias is another limitation because patients were asked to describe past experiences to estimate their QoL at the time of diagnosis and, therefore, caution is advised when interpreting these retrospective data. Because of the online method of survey distribution to patients, it was not possible to estimate the response rate ie, how many patients declined to complete the survey. Nonetheless, I‐WISh has been able to extensively explore the patientʼs perspective of living with ITP and identify those elements that are most important to the patient providing real‐world insight into living with ITP. Discovering the impact on work and fear of dying are among those which are novel and important; however, it was reassuring that the goals of treatment were well‐aligned between patients and experienced physicians who concordantly felt that their communications were generally good.

## 5 CONCLUSIONS

Understand ITP has an adverse impact on HRQoL and day‐to‐day functioning for many patients that can be expressed in many ways. The I‐WISh survey provides unique insights into the perspectives of patients with ITP and the physicians who treat them, amplifying understanding of the multifaceted effects of ITP on patients' lives, one example being the burden of work and reduction in its productivity. Issues and concerns highlighted in this survey for both physicians and patients included fluctuating platelet counts, fear of worsening of disease and dying, impaired social and sex lives, and desire to avoid immunosuppression. Further exploration of specific effects of ITP in individual patients is needed so that these findings can be better integrated into management of patients with ITP. Survey data reporting on the patient journey and symptoms associated with ITP will be reported in a separate manuscript (Cooper et al., manuscript in preparation).

## CONFLICT OF INTEREST

N.C. reports honoraria for speaking engagements and advisory boards from Amgen and Novartis. C.K. received honoraria for speaking engagements and consultancy fees paid to PDSA from Amgen, Novartis, and Rigel Pharmaceuticals. S.W. reports advisory work for Novartis. M.M. reports advisory work for Novartis, Sobi and UCB. D.P. received research grants and honoraria from Novartis and Amgen and consultancy for UCB, MedImmune, and ONO Pharmaceutical. W.G. received research grants from Bayer, BMS, and Novartis and honoraria for participation in advisory boards for Amgen and Novartis. D.M.A. received research grants from Novartis, Amgen, and Bristol‐Myers Squibb and worked as a consultant for Amgen, Novartis, Rigel, and Principia. Y.T. reports honoraria and membership of advisory committees for Novartis and honoraria from Chugai and Kyowa‐Kirin. C.S. reports participating in speakers' bureaus for Amgen, advisory boards for Grifols and Gilead, and speakers' bureaus and advisory boards for Shire/Takeda, Novo Nordisk, Bayer, Pfizer, CSL, Roche, Novartis, and Sobi. M.M. reports membership of advisory boards and speaker engagements for Amgen and Novartis. B.L. reports honoraria for consultancy fee paid to Aipit (Italian Association of Immune Thrombocytopenic Purpura) from UCB and Novartis. J.B.B. reports honoraria, membership of a Board of Directors or advisory board, and research funding for Amgen, Novartis, and GSK, research funding from Boehringer Ingelheim, Prophylix Pharma, Protalex, and Rigel Pharmaceuticals, membership of a Board of Directors or advisory board for Momenta Pharmaceuticals, Prophylix Pharma, Protalex, and Rigel Pharmaceuticals, patents and royalties from UptoDate, and participating in a speakers' bureau for Physician Education Resource. J.H. is a full‐time employee of Novartis Pharma AG. T.B. and G.T.‐S. are employees of Adelphi Real World, which has received consultancy fees from Novartis. A.K., S.L., and M.H. have nothing to disclose.

## AUTHOR CONTRIBUTIONS

All authors contributed to the design of this study, raised awareness, recruited patients and interpreted the data; Tom Bailey and Gavin Taylor‐Stokes coordinated data collection and statistical analysis; and all authors critically reviewed the draft and approved the final version for publication.

## Supporting information


**Appendix S1**. Supporting Information.

**Appendix S2**. Supporting Information.

**Appendix S3**. Supporting Information.

**Appendix S4**. Supporting Information.

## Data Availability

Novartis is committed to sharing with qualified external researchers access to patient‐level data and supporting clinical documents from eligible studies. These requests are reviewed and approved by an independent review panel on the basis of scientific merit. All data provided are anonymized to respect the privacy of patients who have participated in the trial, in line with applicable laws and regulations. This trial data availability is in accordance with the criteria and process described on www.clinicalstudydatarequest.com.
