# Supplementary material for: Immune thrombocytopenia (ITP) World Impact Survey (I‐WISh): Impact of ITP on health‐related quality of life
Source: Am J Hematol. 2020 Dec 19;96(2):199–207. doi: 10.1002/ajh.26036 (PMC7898815; doi:10.1002/ajh.26036)
Supplement: Supplementary file 1 — Appendix S1. Supporting Information. [file AJH-96-199-s001.docx]

**ITP Life Quality Index (ILQI)**

The aim of this questionnaire is to measure how much your ITP has affected your life **OVER THE PAST MONTH.** The aim is to try to standardise how, besides bleeding, your ITP affects your life. Please tick one box.

**1. How often has your ITP impacted on your working life or studies?**

□ Never □ Sometimes □ More than half the time □ All the time

□ I am not currently working/studying due to ITP

□ I am not currently working/studying due to other reasons (0)

**2. How often have you taken time off work or education because of your ITP?**

□ Never □ Sometimes □ More than half the time □ All the time

□ I am not currently working/studying due to ITP

□ I am not currently working/studying due to other reasons (0)

**3. How often has your ITP impacted your ability to concentrate on everyday tasks?**

□ Never □ Sometimes □ More than half the time □ All the time

**4. How often has your ITP impacted your social life?**

□ Never □ Sometimes □ More than half the time □ All the time

**5. How often has your ITP impacted your sex life?**

□ Never □ Sometimes □ More than half the time □ All the time □ Not applicable/prefer not to say

**6. How often has your ITP impacted your energy levels?**

□ Never □ Sometimes □ More than half the time □ All the time

**7. How often has your ITP impacted your undertaking of daily tasks?**

□ Never □ Sometimes □ More than half the time □ All the time

**8. How often has your ITP impacted your ability to support people close to you?**

□ Never □ Sometimes □ More than half the time □ All the time

**9. How often has your ITP negatively impacted your hobbies?**

□ Never □ Sometimes □ More than half the time □ All the time

**10. How often has your ITP negatively impacted your normal capacity to exercise?**

□ Never □ Sometimes □ More than half the time □ All the time

**Please check you have answered EVERY question. Thank you**

**KEY:**

Never=1, Sometimes=2, More than half of the time=3, All the time=4,

I am not currently working/studying due to ITP=4

**Missing:** I am not currently working due to other reasons=0, not applicable/prefer not to say=0

**Min score: 7**

**Max score: 40**

Score of 20 or above suggests significantly impaired quality of life

Score of 30 or above suggests severely impaired quality of life
