# Supplementary material for: Immune thrombocytopenia (ITP) World Impact Survey (I‐WISh): Impact of ITP on health‐related quality of life
Source: Am J Hematol. 2020 Dec 19;96(2):199–207. doi: 10.1002/ajh.26036 (PMC7898815; doi:10.1002/ajh.26036)
Supplement: Supplementary file 2 — Appendix S2. Supporting Information. [file AJH-96-199-s002.docx]

PATIENT SCREENER

S1. Have you been diagnosed with Immune thrombocytopenia/Idiopathic thrombocytopenia (ITP)? <ASK ALL>

Please select one answer

| ⭘ | Yes |
| --- | --- |
| ⭘ | No |
| ⭘ | Don’t know |

S2. How old are you? <ASK ALL>

Please enter your age in the box below

| ________ years (age) |
| --- |
| ⭘ 90 years or above |

S3. Have you previously completed the ITP World Impact Survey (I-WISh)? <ASK ALL>

Please select one answer

| ⭘ | Yes |
| --- | --- |
| ⭘ | No |

S4. What country do you live in? <ASK ALL>

Please select one answer

| ⭘ | Canada |
| --- | --- |
| ⭘ | China |
| ⭘ | Colombia |
| ⭘ | Egypt |
| ⭘ | France |
| ⭘ | Germany |
| ⭘ | India |
| ⭘ | Italy |
| ⭘ | Japan |
| ⭘ | Norway |
| ⭘ | Spain |
| ⭘ | Turkey |
| ⭘ | UK |
| ⭘ | USA |
| ⭘ | None of the above |

S5. Please indicate what region you are from… <ASK depending on code selected at S4>

| **CA** | **CN** | **CO** | **EG** | **FR** | **DE** | **IN** | **IT** | **JP** | **NO** | **ES** | **TU** | **UK** | **US** |
| --- | --- | --- | --- | --- | --- | --- | --- | --- | --- | --- | --- | --- | --- |
| Ontario | Huáběi | Antioquia | Greater Cairo | Paris-Région parisienne | Baden-Württemberg | Andhra Pradesh | Nord ovest | Hokkaido | North | Norte | Central Anatolia | Scotland | Northeast |
| Quebec | Dōngběi | Bolivar | Alexandria | Nord-Est | Bayern | Arunachal Pradesh | Nord est | Tohoku | Trøndelag | Noreste | Eastern Anatolia | Wales | Midwest |
| British Columbia | Huádōng | Boyacá | Delta | Nord-Ouest | Berlin | Assam | Centro | Kanto | West | Centro | Southeastern Anatolia | Northern Ireland | South |
| Alberta | Zhōngnán | Cauca | Suez Canal | Sud-Est | Brandenburg | Bihar | Sud e isole | Chubu | South | Levante |  | South West England | West |
| Nova Scotia | Xīnán | Cundinamarca | North Upper Egypt | Sud-Ouest | Bremen | Chhattisgarh |  | Kinki/Kansai | East | Sur |  | North West England |  |
| Saskatchewan | Xīběi | Magdalena | Asyut |  | Hamburg | Goa |  | Chugoku |  |  |  | East Midlands |  |
| Manitoba |  | Panamá | South Upper Egypt |  | Hessen | Gujarat |  | Shikoku |  |  |  | Greater London |  |
| Newfoundland and Labrador |  | Santander |  |  | Mecklenburg-Vorpommern | Haryana |  | Kyushu (inc.Okinawa) |  |  |  | East of England |  |
| New Brunswick |  | Tolima |  |  | Niedersachsen | Hmachal Pradesh |  |  |  |  |  | North East England |  |
| Prince Edward Island |  |  |  |  | Nordrhein | Jammu & Kashmir |  |  |  |  |  | Yorkshire and the Humber |  |
|  |  |  |  |  | Westfalen-Lippe | Jharkhand |  |  |  |  |  | South East England |  |
|  |  |  |  |  | Rheinland-Pfalz | Karnataka |  |  |  |  |  | West Midlands |  |
|  |  |  |  |  | Saarland | Kerla |  |  |  |  |  |  |  |
|  |  |  |  |  | Sachsen | Madhya Pradesh |  |  |  |  |  |  |  |
|  |  |  |  |  | Sachsen-Anhalt | Maharashtra |  |  |  |  |  |  |  |
|  |  |  |  |  | Schleswig-Holstein | Manipur |  |  |  |  |  |  |  |
|  |  |  |  |  | Thüringen | Meghalaya |  |  |  |  |  |  |  |
|  |  |  |  |  |  | Mizoram |  |  |  |  |  |  |  |
|  |  |  |  |  |  | Nagaland |  |  |  |  |  |  |  |
|  |  |  |  |  |  | Odisha |  |  |  |  |  |  |  |
|  |  |  |  |  |  | Punjab |  |  |  |  |  |  |  |
|  |  |  |  |  |  | Rajasthan |  |  |  |  |  |  |  |
|  |  |  |  |  |  | Sikkim |  |  |  |  |  |  |  |
|  |  |  |  |  |  | Tamil Nadu |  |  |  |  |  |  |  |
|  |  |  |  |  |  | Telangana |  |  |  |  |  |  |  |
|  |  |  |  |  |  | Tripura |  |  |  |  |  |  |  |
|  |  |  |  |  |  | Uttarakhand |  |  |  |  |  |  |  |
|  |  |  |  |  |  | Uttar Pradesh |  |  |  |  |  |  |  |
|  |  |  |  |  |  | West Bengal |  |  |  |  |  |  |  |

S6. Where did you initially hear about the I-WISh survey from? <ASK ALL>

| ⭘ | A patient support group |
| --- | --- |
| ⭘ | My doctor |
| ⭘ | Other |

SECTION A: DIAGNOSIS OF ITP (immune thrombocytopenia / idiopathic thrombocytopenia)

Q1. Please specify your sex. <ASK ALL>

| ⭘ | Male |
| --- | --- |
| ⭘ | Female |

Q2. How long have you had ITP? <ASK ALL>

Please enter the time below (in either years and/or months)

| ________ years |
| --- |
| ________ months |

Q3a. Who did you go to see when your ITP symptoms first appeared (before you knew the diagnosis) and you needed to see a medical person? <ASK ALL>

Please select one answer

| ⭘ | GP/Family doctor |
| --- | --- |
| ⭘ | Nurse <SHOW ‘Medical check-up doctor’ in JP not ‘Nurse’> |
| ⭘ | Accident and Emergency / Emergency room doctor |
| ⭘ | Specialist doctor (such as a haematologist) |
| ⭘ | Dentist |
| ⭘ | Other |
| ⭘ | Don’t know / Can’t remember |

Q3b. Did your <insert answer from Q3a> refer you onto another doctor before you were told that you had ITP? <ASK ALL EXCEPT THOSE ANSWERING 6 OR 7 AT Q3a>

Please select one answer

| ⭘ | Yes |
| --- | --- |
| ⭘ | No |

Q3c. To the best of your knowledge, how many medical people (e.g. doctor, nurse, dentist etc.) did you see before being told you had ITP? <ASK TO THOSE SELECTING code 1 ‘Yes’ at AQ3b>

| ________ number of medical people seen before being told that I had ITP |
| --- |

Q3d. Who diagnosed you with ITP? <ASK ALL>

Please select one answer

| ⭘ | GP/Family doctor |
| --- | --- |
| ⭘ | Nurse<SHOW ‘Medical check-up doctor’ in JP not ‘Nurse’> |
| ⭘ | Accident & Emergency / Emergency Room doctor |
| ⭘ | Specialist doctor (such as a haematologist) |
| ⭘ | Dentist |
| ⭘ | Other |
| ⭘ | Don’t know |

Q3e. Please estimate the following: <ASK ALL>

For each row, write the number of weeks, months or years, as appropriate

| 1. Time from first experiencing ITP symptoms to the first time you went to see a medical person? | | | | | | | |
| --- | --- | --- | --- | --- | --- | --- | --- |
| _________ | ⭘ Days | ⭘ Weeks | ⭘ Months | ⭘ Years | ⭘ Don’t know | | ⭘ Did not have symptoms at initial consultation |
| 1. Time from first going to a medical person to the time of the actual diagnosis of ITP | | | | | | | |
| _________ | ⭘ Days | ⭘ Weeks | ⭘ Months | ⭘ Years | | ⭘ Don’t know | |

Q4. Following your initial visit to a medical person (e.g. doctor), did you feel you experienced a long delay in receiving your ITP diagnosis? <ASK ALL>

| ⭘ | Yes |
| --- | --- |
| ⭘ | No <Skip to QA6> |

Q5a. In your own opinion, why do you think there was a delay in your diagnosis of ITP? <ASK IF Q4 = code 1>

Please select all that apply

| 🞎 | The doctor told me to monitor the symptoms to see if they got worse |
| --- | --- |
| 🞎 | I had to wait to be referred to a specialist who could make the diagnosis |
| 🞎 | I had to wait for additional tests conducted so that my doctor could confirm the diagnosis |
| 🞎 | I was first diagnosed with another condition, not ITP |
| 🞎 | My platelet count was on the border of being considered related to ITP |
| 🞎 | Other |
| ⭘ | Don’t know |

Q5b. How anxious were you during this delay in receiving a diagnosis of ITP? <ASK IF Q4 = code 1>

Please select a rating below, where 1 (I did not feel anxious); to 7 (I felt extremely anxious)

| 1  ⭘ | 2  ⭘ | 3  ⭘ | 4  ⭘ | 5  ⭘ | 6  ⭘ | 7  ⭘ |
| --- | --- | --- | --- | --- | --- | --- |

Q6. As far as you are aware, is your ITP the result of another existing condition? <ASK ALL>

Please select one answer

| ⭘ | Yes |
| --- | --- |
| ⭘ | No |
| ⭘ | Don’t know |

Q7a. Following your ITP diagnosis, where did you find support to help you deal with your condition? <ASK ALL>

Please select all answers that apply

| 🞎 | Family/friends |
| --- | --- |
| 🞎 | Patient support group |
| 🞎 | Other patients (not part of an official support group) |
| 🞎 | Physician |
| 🞎 | Nurse |
| 🞎 | Other type of support |
| ⭘ | I did not have any support |

Q7b. Would you have liked to have had more support at the time of your diagnosis? <ASK ALL>

Please select one answer

| ⭘ | Yes |
| --- | --- |
| ⭘ | No |

Q7c. From whom would you have liked to have had more support from to help you deal with your condition? <Ask if AQ7b = code 1>

Please select all answers that apply

| 🞎 | Family/friends |
| --- | --- |
| 🞎 | Patient support group |
| 🞎 | Other patients (not part of an official group) |
| 🞎 | Physician |
| 🞎 | Nurse |
| 🞎 | Other type of support |

END OF SECTION

SECTION B: SYMPTOMS

Q1. How would you describe your current health? <ASK ALL>

Please select a rating below, from 1 (very poor health) to 7 (excellent health)

| 1  ⭘ | 2  ⭘ | 3  ⭘ | 4  ⭘ | 5  ⭘ | 6  ⭘ | 7  ⭘ |
| --- | --- | --- | --- | --- | --- | --- |

Q2. Which of the following symptoms, if any, did/do you have…<ASK ALL>

1. At the time of your ITP diagnosis?
2. Currently?

Please select all that apply

|  |  | a) At the time of your ITP diagnosis? | b) Currently? |
| --- | --- | --- | --- |
| 1 | Bruising or purplish areas on the skin or mucous membranes (such as the mouth) occurring for no known reason | 🞎 | 🞎 |
| 2 | Petechiae – pinpoint red spots on the skin often found in groups and could appear as a rash and do not feel like a raised lump when you touch them and do not itch | 🞎 | 🞎 |
| 3 | Haematoma – a collection of clotted or partially clotted blood under the skin that looks or feels like a lump or bump. | 🞎 | 🞎 |
| 4 | Thrombosis – formation of a blood clot within a blood vessel blocking the flow in that vessel (must be enough for your doctor to take it seriously and discuss it with you) | 🞎 | 🞎 |
| 5 | Spontaneous nosebleeds (ones occurring with no reason) | 🞎 | 🞎 |
| 6 | Prolonged or unusually heavy bleeding from the gums (e.g. with tooth brushing, flossing or during dental work) | 🞎 | 🞎 |
| 7 | Prolonged bleeding from cuts | 🞎 | 🞎 |
| 8 | Extra bleeding during or immediately after surgery | 🞎 | 🞎 |
| 9 | Blood in the urine or stool (bowel movement) | 🞎 | 🞎 |
| 10 | Heavy menstrual bleeding either increased bleeding during days of bleeding or heavy bleeding occurring for more days than usual <Only if AQ1 = code 2> | 🞎 | 🞎 |
| 11 | Fatigue (tiredness, lack of energy) | 🞎 | 🞎 |
| 12 | Increased numbers of moderate to severe headaches/migraines | 🞎 | 🞎 |
| 13 | Dizziness | 🞎 | 🞎 |
| 14 | Depression | 🞎 | 🞎 |
| 15 | Anxiety surrounding unstable platelet count | 🞎 | 🞎 |
| 16 | Other | 🞎 | 🞎 |
| 17 | No symptoms at diagnosis | ⭘ | ⭘ |

Q3a. Of the symptoms you had at **diagnosis**, how would you have rated the severity of these at the time? <ASK ALL, ONLY SHOW SYMPTOMS SELECTED AT BQ2a>

Please rate each symptom below, where 1 (not severe at all); to 7 (worst imaginable).

|  |  | 1 | 2 | 3 | 4 | 5 | 6 | 7 |
| --- | --- | --- | --- | --- | --- | --- | --- | --- |
| 1 | Bruising or purplish areas on the skin or mucous membranes (such as the mouth) occurring for no known reason | ⭘ | ⭘ | ⭘ | ⭘ | ⭘ | ⭘ | ⭘ |
| 2 | Petechiae – pinpoint red spots on the skin often found in groups and could appear as a rash and do not feel like a raised lump when you touch them and do not itch | ⭘ | ⭘ | ⭘ | ⭘ | ⭘ | ⭘ | ⭘ |
| 3 | Haematoma – a collection of clotted or partially clotted blood under the skin that looks or feels like a lump or bump. | ⭘ | ⭘ | ⭘ | ⭘ | ⭘ | ⭘ | ⭘ |
| 4 | Thrombosis – formation of a blood clot within a blood vessel blocking the flow in that vessel (must be enough for your doctor to take it seriously and discuss it with you) | ⭘ | ⭘ | ⭘ | ⭘ | ⭘ | ⭘ | ⭘ |
| 5 | Spontaneous nosebleeds (ones occurring with no reason) | ⭘ | ⭘ | ⭘ | ⭘ | ⭘ | ⭘ | ⭘ |
| 6 | Prolonged or unusually heavy bleeding from the gums (e.g. with tooth brushing, flossing or during dental work) | ⭘ | ⭘ | ⭘ | ⭘ | ⭘ | ⭘ | ⭘ |
| 7 | Prolonged bleeding from cuts | ⭘ | ⭘ | ⭘ | ⭘ | ⭘ | ⭘ | ⭘ |
| 8 | Extra bleeding during or immediately after surgery | ⭘ | ⭘ | ⭘ | ⭘ | ⭘ | ⭘ | ⭘ |
| 9 | Blood in the urine or stool (bowel movement) | ⭘ | ⭘ | ⭘ | ⭘ | ⭘ | ⭘ | ⭘ |
| 10 | Heavy menstrual bleeding either increased bleeding during days of bleeding or heavy bleeding occurring for more days than usual <Only if AQ1 = code 2> | ⭘ | ⭘ | ⭘ | ⭘ | ⭘ | ⭘ | ⭘ |
| 11 | Fatigue (tiredness, lack of energy) | ⭘ | ⭘ | ⭘ | ⭘ | ⭘ | ⭘ | ⭘ |
| 12 | Increased numbers of moderate to severe Headaches/migraines | ⭘ | ⭘ | ⭘ | ⭘ | ⭘ | ⭘ | ⭘ |
| 13 | Dizziness | ⭘ | ⭘ | ⭘ | ⭘ | ⭘ | ⭘ | ⭘ |
| 14 | Depression | ⭘ | ⭘ | ⭘ | ⭘ | ⭘ | ⭘ | ⭘ |
| 15 | Anxiety surrounding unstable platelet count | ⭘ | ⭘ | ⭘ | ⭘ | ⭘ | ⭘ | ⭘ |

Q3b. Of the symptoms you **currently** experience, how would you rate the severity of these? <ASK ALL, ONLY SHOW SYMPTOMS SELECTED AT BQ2b>

Please rate each symptom below, where 1 (not severe at all) to 7 (worst imaginable).

|  |  | 1 | 2 | 3 | 4 | 5 | 6 | 7 |
| --- | --- | --- | --- | --- | --- | --- | --- | --- |
| 1 | Bruising or purplish areas on the skin or mucous membranes (such as the mouth) occurring for no known reason | ⭘ | ⭘ | ⭘ | ⭘ | ⭘ | ⭘ | ⭘ |
| 2 | Petechiae – pinpoint red spots on the skin often found in groups and could appear as a rash and do not feel like a raised lump when you touch them and do not itch | ⭘ | ⭘ | ⭘ | ⭘ | ⭘ | ⭘ | ⭘ |
| 3 | Haematoma – a collection of clotted or partially clotted blood under the skin that looks or feels like a lump or bump. | ⭘ | ⭘ | ⭘ | ⭘ | ⭘ | ⭘ | ⭘ |
| 4 | Thrombosis – formation of a blood clot within a blood vessel blocking the flow in that vessel (must be enough for your doctor to take it seriously and discuss it with you) | ⭘ | ⭘ | ⭘ | ⭘ | ⭘ | ⭘ | ⭘ |
| 5 | Spontaneous nosebleeds (ones occurring with no reason) | ⭘ | ⭘ | ⭘ | ⭘ | ⭘ | ⭘ | ⭘ |
| 6 | Prolonged or unusually heavy bleeding from the gums (e.g. with tooth brushing, flossing or during dental work) | ⭘ | ⭘ | ⭘ | ⭘ | ⭘ | ⭘ | ⭘ |
| 7 | Prolonged bleeding from cuts | ⭘ | ⭘ | ⭘ | ⭘ | ⭘ | ⭘ | ⭘ |
| 8 | Extra bleeding during or immediately after surgery | ⭘ | ⭘ | ⭘ | ⭘ | ⭘ | ⭘ | ⭘ |
| 9 | Blood in the urine or stool (bowel movement) | ⭘ | ⭘ | ⭘ | ⭘ | ⭘ | ⭘ | ⭘ |
| 10 | Heavy menstrual bleeding either increased bleeding during days of bleeding or heavy bleeding occurring for more days than usual <Only if AQ1 = code 2> | ⭘ | ⭘ | ⭘ | ⭘ | ⭘ | ⭘ | ⭘ |
| 11 | Fatigue (tiredness, lack of energy) | ⭘ | ⭘ | ⭘ | ⭘ | ⭘ | ⭘ | ⭘ |
| 12 | Increased numbers of moderate to severe Headaches/migraines | ⭘ | ⭘ | ⭘ | ⭘ | ⭘ | ⭘ | ⭘ |
| 13 | Dizziness | ⭘ | ⭘ | ⭘ | ⭘ | ⭘ | ⭘ | ⭘ |
| 14 | Depression | ⭘ | ⭘ | ⭘ | ⭘ | ⭘ | ⭘ | ⭘ |
| 15 | Anxiety surrounding unstable platelet count | ⭘ | ⭘ | ⭘ | ⭘ | ⭘ | ⭘ | ⭘ |

Q4. Which 3 current symptoms would you most want to be made better or resolved? <ASK ALL, ONLY SHOW SYMPTOMS SELECTED AT BQ2b>

Please assign rankings (1 to 3) with 1 being the symptom you would most like to be made better or resolved

| 1 | 🞎 | Purpura – bruising or purplish areas on the skin or mucous membranes (such as the mouth) occurring for no known reason |
| --- | --- | --- |
| 2 | 🞎 | Petechiae – pinpoint red spots on the skin often found in groups and could appear as a rash |
| 3 | 🞎 | Haematoma – a collection of clotted or partially clotted blood under the skin that looks or feels like a lump. |
| 4 | 🞎 | Thrombosis – formation of a blood clot within a blood vessel. |
| 5 | 🞎 | Spontaneous nosebleeds |
| 6 | 🞎 | Bleeding from the gums (e.g. during dental work) |
| 7 | 🞎 | Prolonged bleeding from cuts |
| 8 | 🞎 | Profuse bleeding during surgery |
| 9 | 🞎 | Blood in the urine or stool (bowel movement) |
| 10 | 🞎 | Heavy menstrual bleeding <Only if AQ1 = code 2> |
| 11 | 🞎 | Fatigue (tiredness) |
| 12 | 🞎 | Headaches/migraines |
| 13 | 🞎 | Dizziness |

SECTION C: ITP LIFE QUALITY INDEX (ILQI)

The aim of this section is to measure how much your ITP has affected your life **OVER THE PAST MONTH.** The aim is to try to standardise how, besides bleeding, your ITP affects your life. Please tick one box.

1. **How often has your ITP impacted on your working life or studies?**

□ Never □ Sometimes □ More than half the time □ All the time

□ I am not currently working/studying due to ITP

□ I am not currently working/studying due to other reasons (0)

1. **How often have you taken time off work or education because of your ITP?**

□ Never □ Sometimes □ More than half the time □ All the time

□ I am not currently working/studying due to ITP

□ I am not currently working/studying due to other reasons (0)

1. **How often has your ITP impacted your ability to concentrate on everyday tasks?**

□ Never □ Sometimes □ More than half the time □ All the time

1. **How often has your ITP impacted your social life?**

□ Never □ Sometimes □ More than half the time □ All the time

1. **How often has your ITP impacted your sex life?**

□ Never □ Sometimes □ More than half the time □ All the time

□ Not applicable/prefer not to say

1. **How often has your ITP impacted your energy levels?**

□ Never □ Sometimes □ More than half the time □ All the time

1. **How often has your ITP impacted your undertaking of daily tasks?**

□ Never □ Sometimes □ More than half the time □ All the time

1. **How often has your ITP impacted your ability to support people close to you?**

□ Never □ Sometimes □ More than half the time □ All the time

1. **How often has your ITP negatively impacted your hobbies?**

□ Never □ Sometimes □ More than half the time □ All the time

1. **How often has your ITP negatively impacted your normal capacity to exercise?**

□ Never □ Sometimes □ More than half the time □ All the time

Q11. Thinking of how your ITP condition affects you emotionally, please rate the following statements where 1 = not at all and 7 = a great deal: <ASK ALL>

|  |  | 1  not at all | 2 | 3 | 4 | 5 | 6 | 7  a great deal |
| --- | --- | --- | --- | --- | --- | --- | --- | --- |
| 1 | I often feel anxious/nervous about my platelet counts | ⭘ | ⭘ | ⭘ | ⭘ | ⭘ | ⭘ | ⭘ |
| 2 | I feel frustrated by having to put up with the symptoms of my disease | ⭘ | ⭘ | ⭘ | ⭘ | ⭘ | ⭘ | ⭘ |
| 3 | I worry that my condition will get worse | ⭘ | ⭘ | ⭘ | ⭘ | ⭘ | ⭘ | ⭘ |
| 4 | I am stressed about my ITP condition | ⭘ | ⭘ | ⭘ | ⭘ | ⭘ | ⭘ | ⭘ |
| 5 | I feel helpless because of my ITP | ⭘ | ⭘ | ⭘ | ⭘ | ⭘ | ⭘ | ⭘ |
| 6 | I worry about dying | ⭘ | ⭘ | ⭘ | ⭘ | ⭘ | ⭘ | ⭘ |
| 7 | I am worried that I often feel worse than my doctor thinks I feel | ⭘ | ⭘ | ⭘ | ⭘ | ⭘ | ⭘ | ⭘ |
| 8 | I often worry about my physical appearance (i.e. bruising, rashes, lumps) | ⭘ | ⭘ | ⭘ | ⭘ | ⭘ | ⭘ | ⭘ |
| 9 | I often wear long sleeved clothing, even when it is warm, to hide the signs of bleeding | ⭘ | ⭘ | ⭘ | ⭘ | ⭘ | ⭘ | ⭘ |
| 10 | I worry about my disease ( i.e. I am fearful of major bleeds, either in my head or internal bleeding in my stomach) | ⭘ | ⭘ | ⭘ | ⭘ | ⭘ | ⭘ | ⭘ |
| 11 | It is important to me that I have a stable and safe platelet count | ⭘ | ⭘ | ⭘ | ⭘ | ⭘ | ⭘ | ⭘ |
| 12 | I worry that my platelet count goes up and down for no apparent reason | ⭘ | ⭘ | ⭘ | ⭘ | ⭘ | ⭘ | ⭘ |

Q12a. Overall, how does your ITP impact on your emotional well-being (e.g. stress, anxiety, mood)? <ASK ALL>

Please rate each statement below, where 1 = not at all and 7 = a great deal.

| 1  not at all | 2 | 3 | 4 | 5 | 6 | 7  a great deal |
| --- | --- | --- | --- | --- | --- | --- |
| ⭘ | ⭘ | ⭘ | ⭘ | ⭘ | ⭘ | ⭘ |

Q12b. Do you get any professional support (e.g. psychiatrist, counselling support) to help you manage with the emotional aspects of your ITP (e.g. stress, anxiety, mood)? <ASK ALL>

| ⭘ | Yes |
| --- | --- |
| ⭘ | No |

Q12c. Do you wish you were able to receive additional professional support (e.g. psychiatrist, counselling support) for the emotional aspects of your ITP (e.g. stress, anxiety, mood)? <ASK IF CQ12b = code 2>

| ⭘ | Yes |
| --- | --- |
| ⭘ | No |

SECTION D: IMPACT ON WORK, FINANCES AND SUPPORT

Q1. As a result of your ITP, have you ever… <ASK ALL>

Please select all that apply

|  | **Yes** | **No** | **Not applicable** |
| --- | --- | --- | --- |
| Changed from full-time to part-time employment? | ⭘ | ⭘ | ⭘ |
| Reduced your hours at work? | ⭘ | ⭘ | ⭘ |
| Seriously considered reducing your hours at work (even if you did not)? | ⭘ | ⭘ | ⭘ |
| Taken early retirement? | ⭘ | ⭘ | ⭘ |
| Voluntarily stopped working in your job? | ⭘ | ⭘ | ⭘ |
| Been sacked or made to stop working in your job? | ⭘ | ⭘ | ⭘ |
| Considered terminating your job (even if you did not)? | ⭘ | ⭘ | ⭘ |
| Declined a promotion? | ⭘ | ⭘ | ⭘ |

Q2. (WPAI Q1) Are you currently employed (working for pay)? <ASK ALL>

| ⭘ | Yes |
| --- | --- |
| ⭘ | No <Go to DQ7> |

| Q type | Single code | VAR |  | ARW STANDARD |
| --- | --- | --- | --- | --- |
| Range |  | | | |
| Base | All | | | |
| Logic | Code 2 “No” Skip to DQ7 | | | |

Q3. (WPAI Q2) During the past seven days, how many hours did you miss from work because of problems associated with your ITP condition? *Include hours you missed on sick days, times you went in late, left early, etc. because of your ITP. Do not include time you missed to participate in this study.*

| ________ Hours |
| --- |

| Range | 0-168 |
| --- | --- |

Q4. (WPAI Q3) During the past seven days, how many hours did you miss from work because of any other reason, such as vacation, holidays, time off to participate in this study?

| ________ Hours |
| --- |

| Range | 0-168 |
| --- | --- |

Q5. (WPAI Q4) During the past seven days, how many hours did you actually work?

Please enter the number of hours

| ________ Hours |
| --- |

Q6. (WPAI Q5) During the past 7 days, how much did your ITP condition affect your productivity while you were working? *Think about days you were limited in the amount or kind of work you could do, days you accomplished less than you would like, or days you could not do your work as carefully as usual because of your ITP. If ITP affected your work only a little, choose a low number. Choose a high number if ITP affected your work a great deal.* Consider only how much your ITP condition affected productivity while you were working.

Please use the rating scale below

| 1  ⭘  ITP had no effect on my work | 2  ⭘ | 3  ⭘ | 4  ⭘ | 5  ⭘ | 6  ⭘ | 7  ⭘ | 8  ⭘ | 9  ⭘ | 10  ⭘  ITP completely prevented me from working |
| --- | --- | --- | --- | --- | --- | --- | --- | --- | --- |

Q7. <ASK ALL> (WPAI Q6) During the past 7 days, how much did your ITP condition affect your ability to do your regular daily activities, other than work at a job? *By regular activities, we mean the usual activities you do such as work around the house, shopping, childcare, exercising, studying, etc. Think about times you were limited in the amount or kind of activities you could do and times you accomplished less than you would like. If ITP affected your activities only a little, choose a low number. Choose a high number if ITP affected your activities a great deal.*

Please use the rating scale below

| 1  ⭘  ITP had no effect on my daily activities | 2  ⭘ | 3  ⭘ | 4  ⭘ | 5  ⭘ | 6  ⭘ | 7  ⭘ | 8  ⭘ | 9  ⭘ | 10  ⭘  ITP completely prevented me from doing my daily activities |
| --- | --- | --- | --- | --- | --- | --- | --- | --- | --- |

Q8. What is your current employment status? <If DQ2 = code 1 only show code 1 and 2. If DQ2 = code 2 don’t show code 1 and 2>

Please select one answer

| ⭘ | Working full-time |
| --- | --- |
| ⭘ | Working part-time |
| ⭘ | Not working seeking employment |
| ⭘ | Not working not seeking employment |
| ⭘ | Retired |
| ⭘ | Homemaker |
| ⭘ | Student |
| ⭘ | On Disability |
| ⭘ | On long term sick leave |
| ⭘ | Other |

Q9. What best describes your type of health insurance coverage? <ASK ALL, SHOW COUNTRY SPECIFIC OPTIONS>

Please select one answer

| Canada | China | Colombia | Egypt | France | India | Italy | Germany | Japan | Norway | Spain | Turkey | UK | US |
| --- | --- | --- | --- | --- | --- | --- | --- | --- | --- | --- | --- | --- | --- |
| ⭘ Public drug plan (provided through provincial or territorial health plan) | ⭘ Urban Employee Basic Medical Insurance | ⭘ Contributive regime (POS-C) | ⭘ Public insurance schemes | ⭘ Protection Universelle Maladie (PUMa) | ⭘ National/Social Health Insurance Schemes | ⭘ Servizio Sanitario Nazionale | ⭘ Gesetzliche Krankenversicherung (GKV) | ⭘ Employee provided for a small/medium enterprise (less than 300 employees) | ⭘ Folketrygden, NIS | ⭘ Sistema Nacional de Salud (SNS) | ⭘ Universal health Insurance | ⭘ NHS | ⭘ Medicare |
| ⭘ Employer provided/sponsored insurance | ⭘ Urban Resident Basic Medical Insurance | ⭘ Subsidized regime (POS-S) | ⭘ Private insurance schemes | ⭘ PMUa + CMU-C, mutuelle or assurance privée | ⭘ Rashtiya Swasthiya Bima Yojana (RSBY) | ⭘ Servizio Sanitario Nazionale & Assicurazione sanitaria privata | ⭘ Private Krankenversicherung (PKV) | ⭘ Employee provided for a large enterprise (more than 300 enterprises) | ⭘ Private insurance | ⭘ Seguro médico privado | ⭘ Voluntary, private health insurance | ⭘ Private self-paid | ⭘ Medicaid (or equivalent in your state) |
| ⭘ No insurance coverage | ⭘ New Rural Cooperative Medical Insurance | ⭘ Special regime (i.e. teacher or military) | ⭘ Other | ⭘ Patient non assuré | ⭘ Employment State Insurance Scheme (ESIS) | ⭘ Assicurazione sanitaria privata | ⭘Andere | ⭘ Mutual aid association (for public servants, teachers) | ⭘ Other | ⭘ Sistema Nacional de Salud (SNS), y seguro médico privado | ⭘ No insurance coverage | ⭘ Private insurance covered | ⭘ Medicare part D prescription drug plan |
| ⭘ Other | ⭘ Medical assistance program | ⭘ No insurance coverage | ⭘ Don’t know | ⭘ Autre | ⭘ Central Government Health Scheme (CGHS) | ⭘ Altro | ⭘ Keine | ⭘ Seaman's Insurance | ⭘ Don’t know | ⭘ El paciente mismo | ⭘ Other | ⭘ Other | ⭘ Medicare medical savings account (MSA) |
| ⭘ Don’t know | ⭘ Private health insurance (including prescription drug cover) | ⭘ Other | ⭘ No insurance | ⭘ Ne sait pas | ⭘ Aam Aadmi Bima Yojana (AABY) | ⭘ No lo so | ⭘ Weiß nicht | ⭘ National Health Insurance provided by municipality (for self-employed, unemployed, students, retired and farm workers) |  | ⭘ Sin seguro médico | ⭘ Don’t know | ⭘ Don’t know | ⭘ Medicare advantage |
|  | ⭘ Private health insurance (excluding prescription drug cover) | ⭘ Don’t know |  |  | ⭘ Janashree Bima Yojana (JBY) | ⭘ Non saprei |  | ⭘ Health insurance for the elderly (for over 75 years) |  | ⭘ Otro |  |  | ⭘ Employer provided/sponsored insurance |
|  | ⭘ No coverage |  |  |  | ⭘ Universal Health Insurance Scheme (UHIS) |  |  | ⭘ Private, supplementary health insurance |  | ⭘ No sé |  |  | ⭘ Partner/family member employer insurance |
|  | ⭘ Other |  |  |  | ⭘ State Health Insurance Programmes |  |  | ⭘ No insurance |  |  |  |  | ⭘ Privately arranged insurance |
|  | ⭘ Don’t know |  |  |  | ⭘ Rajiv Aarogyasri, Andhra Pradesh |  |  | ⭘ Other |  |  |  |  | ⭘ Health insurance exchange plan |
|  |  |  |  |  | ⭘ Mukhyamantari Amrutam (MA), Gujarat |  |  | ⭘ Don’t know |  |  |  |  | ⭘ Cobra (continuation coverage) |
|  |  |  |  |  | ⭘ The Chief Minister's Distress Relied Fund, Kerala |  |  |  |  |  |  |  | ⭘ Non-Medicare retired benefit |
|  |  |  |  |  | ⭘ Chief Minister's Relief Fund, Madhya Pradesh |  |  |  |  |  |  |  | ⭘ Tricare/Veterans health care |
|  |  |  |  |  | ⭘ Rajasthan's Chief Minister's Relief fund, Rajasthan |  |  |  |  |  |  |  | ⭘ No insurance coverage |
|  |  |  |  |  | ⭘ Chief Minister's Comprehensive Health Insurance Scheme, Tamil Nadu |  |  |  |  |  |  |  | ⭘ Other |
|  |  |  |  |  | ⭘ Private health insurance |  |  |  |  |  |  |  | ⭘ Don’t know |
|  |  |  |  |  | ⭘ No insurance |  |  |  |  |  |  |  |  |
|  |  |  |  |  | ⭘ Other |  |  |  |  |  |  |  |  |
|  |  |  |  |  | ⭘ Don’t know |  |  |  |  |  |  |  |  |

Q10a. In an average month, how much do you pay out of your own money (excluding any money you may receive to support your disease, i.e. from your government) for the below expenses, in relation to your ITP condition? <ASK ALL>

Please select one answer

| **Type of expense** | **Approximate cost (*<currency>*/monthly)** |
| --- | --- |
| Daily assistance (i.e. from a nurse or paid caregiver) | ______________ |
| Medicines (including both prescription and non-prescription medicines) | ______________ |
| Travel to doctor/hospital appointments | ______________ |
| Tests, i.e. blood tests, scans, etc. | ______________ |
| Parking fees (i.e. at the pharmacy, or hospital/clinic) | ______________ |
| Other costs incurred | ______________ |

Q10b. In an average month, how long do you spend traveling to and from appointments in relation to you ITP condition? <ASK ALL>

| ________ hours |
| --- |

Q11. How often do you require support from someone to assist you with your activities of daily living due to your ITP? <ASK ALL>

*Support can include assistance with activities of daily living, ranging from a few hours of shopping and cleaning to intensive medical or personal care. Tasks can include shopping, house cleaning, cooking, giving medications, assistance going to the toilet and so forth.*

Please select one answer

| ⭘ | Never <skip to DQ15> |
| --- | --- |
| ⭘ | Rarely |
| ⭘ | Sometimes |
| ⭘ | Often |

Q12. Who is the main person who provides you with this support?

Please select one answer

| ⭘ | Spouse/partner |
| --- | --- |
| ⭘ | Son/daughter |
| ⭘ | Parent |
| ⭘ | Sibling / Other relative |
| ⭘ | Friend / Neighbour |
| ⭘ | Professional caregiver (i.e. a person paid to care for you) |

Q13. What kind of help/support do you require from your <INSERT ANSWER FROM DQ12> as a result of your ITP?

Please select all that apply

| 🞎 | Companionship (e.g. talking, reading, keeping company) supervision, emotional support or encouragement |
| --- | --- |
| 🞎 | Transportation (e.g. driving to doctor’s appointments, driving for errands) |
| 🞎 | Homemaking (e.g. shopping, cleaning, preparing meals) |
| 🞎 | Personal care assistance (e.g. feeding, bathing, toileting, dressing, grooming) |
| 🞎 | Healthcare assistance (e.g. help with medications, wound care, learning more about the condition) |
| 🞎 | Managing finances (e.g. paying bills, managing budget) |
| 🞎 | Help plan and organize everyday activities |
| 🞎 | Other |

Q14. In the past 7 days, how many hours help/support have you received from your <INSERT ANSWER FROM DQ12> for the activities you listed previously, for your ITP condition?

Please enter the number of hours

| ________ Hours |
| --- |

Q15. To what extent does ITP interfere with the following aspects of your life? <ASK ALL>

Please rate the following options, where 1 (not at all); to 7 (a great deal)

|  |  | 1  not at all | 2 | 3 | 4 | 5 | 6 | 7  a great deal | Not applicable |
| --- | --- | --- | --- | --- | --- | --- | --- | --- | --- |
| 1 | Daily activities (i.e. food preparation, housework, gardening, taking care of children, oral hygiene) | ⭘ | ⭘ | ⭘ | ⭘ | ⭘ | ⭘ | ⭘ | ⭘ |
| 2 | Family or social life (meeting friends/family for activities, hobbies) | ⭘ | ⭘ | ⭘ | ⭘ | ⭘ | ⭘ | ⭘ | ⭘ |
| 3 | Relationship with (informal and formal) caregiver <ASK TO THOSE SELECTING code 2-4 at DQ11> | ⭘ | ⭘ | ⭘ | ⭘ | ⭘ | ⭘ | ⭘ | ⭘ |
| 4 | Relationship with spouse/partner | ⭘ | ⭘ | ⭘ | ⭘ | ⭘ | ⭘ | ⭘ | ⭘ |

END OF SECTION

SECTION E: TREATMENT OF ITP

Q1.If you required treatment, how long did you have to wait after your ITP diagnosis to receive treatment for your ITP? (e.g. drug treatment, splenectomy, platelet/blood transfusion) <ASK ALL>

Please enter a number as relevant below

|  | ________ weeks |
| --- | --- |
|  | ________ months |
|  | ________ years |
| ⭘ | Not yet received any treatment for my ITP |
| ⭘ | Received treatment straight away |

Range for years: 0-10, months: 0-36, weeks :0-52

At least one row must be completed; auto populate with zeros if code 3 selected, or only code1/2 selected.

Q2. Have you ever been on ‘watch and wait’ management by your doctor? (i.e. received no medical treatments for a period of time because it was felt that treatment was not required) <ASK ALL>

Please select one answer below

| ⭘ | Yes |
| --- | --- |
| ⭘ | No |
| ⭘ | Don’t know |

Q3a. Do you feel as though your symptoms (e.g. fatigue/energy levels) and your quality of life were taken into account by your treating doctor when deciding if you needed treatment? <ASK ALL>

Please select one answer below

| ⭘ | Yes |
| --- | --- |
| ⭘ | No |
| ⭘ | Don’t know |

Q3b. Do you wish that your symptoms and quality of life were taken into account when this treatment decision was made? <ASK TO THOSE WHO SELECT CODE 2 AT EQ3a>

Please select one answer below

| ⭘ | Yes |
| --- | --- |
| ⭘ | No |

Q4a. Thinking about the monitoring of your ITP, do you feel that your quality of life and symptoms (e.g. fatigue/energy levels) have been assessed in combination with your platelet count and your need for treatment? <ASK ALL>

Please select one answer below

| ⭘ | Yes |
| --- | --- |
| ⭘ | No |
| ⭘ | Don’t know |

Q4b. Do you wish that your platelet count, symptoms and quality of life were taken into account when monitoring your ITP? <ASK TO THOSE WHO SELECT CODE 2 AT EQ4a>

Please select one answer below

| ⭘ | Yes |
| --- | --- |
| ⭘ | No |

<IF EQ1a = NOT YET RECEIVED ANY TREATMENT FOR MY ITP, SKIP TO EQ14a AFTER ANSWERING Q4b>

Q5a. Have you ever undergone a splenectomy? <ASK ALL>

Please select one answer below

| ⭘ | Yes |
| --- | --- |
| ⭘ | No <SKIP TO Q6> |

Q5b. Did you experience any of the following as a consequence of undergoing a splenectomy? <ASK IF EQ5a = code 1>

| 1 | 🞎 Inflammation of the pancreas and/or belly pain |
| --- | --- |
| 2 | 🞎 Frequent Respiratory symptoms |
| 3 | 🞎 Daily need for antibiotics |
| 4 | 🞎 Increased infections of any type |
| 5 | 🞎 Need for regular immunisations |
| 6 | 🞎 Need to carry splenectomy card/wear bracelet when travelling |
| 7 | 🞎 Episodes of sepsis or fever requiring emergency room visits |
| 8 | 🞎 Thrombosis (like a clot in the leg or a pulmonary embolus) |
| 9 | 🞎 Other |
| 10 | ⭘ None of these |

Q5c. How much do you agree with the following statements in relation to your having had splenectomy for your ITP condition? <ASK IF EQ5a = code 1>

Please rate each statement below, where 1 (strongly disagree); to 7 (strongly agree) or D/K if you don’t know or unsure

|  |  | 1 | 2 | 3 | 4 | 5 | 6 | 7 | D/K |
| --- | --- | --- | --- | --- | --- | --- | --- | --- | --- |
| 1 | Overall, I am very satisfied with having had a splenectomy for control of my ITP | ⭘ | ⭘ | ⭘ | ⭘ | ⭘ | ⭘ | ⭘ | ⭘ |
| 2 | Splenectomy has been effective in treating my ITP symptoms | ⭘ | ⭘ | ⭘ | ⭘ | ⭘ | ⭘ | ⭘ | ⭘ |
| 3 | Splenectomy has increased my energy levels | ⭘ | ⭘ | ⭘ | ⭘ | ⭘ | ⭘ | ⭘ | ⭘ |
| 4 | I am worried about the short term side effects of a splenectomy | ⭘ | ⭘ | ⭘ | ⭘ | ⭘ | ⭘ | ⭘ | ⭘ |
| 5 | I worry about the long term side effects of a splenectomy | ⭘ | ⭘ | ⭘ | ⭘ | ⭘ | ⭘ | ⭘ | ⭘ |
| 6 | I would recommend a splenectomy to another ITP patient | ⭘ | ⭘ | ⭘ | ⭘ | ⭘ | ⭘ | ⭘ | ⭘ |
| 7 | I am worried about the immunosuppressive effect of the splenectomy | ⭘ | ⭘ | ⭘ | ⭘ | ⭘ | ⭘ | ⭘ | ⭘ |
| 8 | I regret having had the splenectomy because it did not work | ⭘ | ⭘ | ⭘ | ⭘ | ⭘ | ⭘ | ⭘ | ⭘ |
| 9 | I regret having had the splenectomy even though it worked | ⭘ | ⭘ | ⭘ | ⭘ | ⭘ | ⭘ | ⭘ | ⭘ |
| 10 | I regret having had the splenectomy because it is causing me other problems | ⭘ | ⭘ | ⭘ | ⭘ | ⭘ | ⭘ | ⭘ | ⭘ |
| 11 | Before surgery, I was made aware of the long term complications associated with splenectomy | ⭘ | ⭘ | ⭘ | ⭘ | ⭘ | ⭘ | ⭘ | ⭘ |
| 12 | I am worried about the possible risk of septicaemia as a result of my splenectomy. | ⭘ | ⭘ | ⭘ | ⭘ | ⭘ | ⭘ | ⭘ | ⭘ |
| 13 | I am worried about the side-effects of taking daily oral antibiotics after splenectomy | ⭘ | ⭘ | ⭘ | ⭘ | ⭘ | ⭘ | ⭘ | ⭘ |
| 14 | I am worried about the need for regular immunizations following my splenectomy | ⭘ | ⭘ | ⭘ | ⭘ | ⭘ | ⭘ | ⭘ | ⭘ |
| 15 | I am worried about the increased risk of blood clots following splenectomy | ⭘ | ⭘ | ⭘ | ⭘ | ⭘ | ⭘ | ⭘ | ⭘ |

Q6a. Please select all treatments you have **ever** received to help manage your ITP <ASK ALL>

Please select all that apply

| 1 | 🞎 | Androgens (such as danazol) |
| --- | --- | --- |
| 2 | 🞎 | Anti-CD20 (such as rituximab, veltuzumab or ofatumumab) |
| 3 | 🞎 | Anti-fibrinolytic (such as tranexamic acid or aminocaproic acid) |
| 4 | 🞎 | Other immunosuppressants (such as azathioprine, cyclophosphamide or mofetil) |
| 5 | 🞎 | Cyclosporine (such as cyclosporine A or cytoxin) |
| 6 | 🞎 | Intravenous Immunoglobulins (IVIg) |
| 7 | 🞎 | Platelet transfusion |
| 8 | 🞎 | Rho(D) immune globulin (Anti-D) |
| 9 | 🞎 | Corticosteroids / steroids (such as prednisolone, methylprednisolone or dexamethasone) |
| 10 | 🞎 | Thrombopoietin receptor agonists (such as romiplostim or eltrombopag) |
| 11 | 🞎 | Other |
| 12 | 🞎 | Can’t remember the name of treatment |
| 13 | ⭘ | No therapy |

Q6b. Please select all treatments you are **currently** receiving to help manage your ITP <ASK ALL>

Please select all that apply

| 1 | 🞎 | Androgens (such as danazol) |
| --- | --- | --- |
| 2 | 🞎 | Anti-CD20 (such as rituximab, veltuzumab or ofatumumab) |
| 3 | 🞎 | Anti-fibrinolytic (such as tranexamic acid or aminocaproic acid) |
| 4 | 🞎 | Other immunosuppressants (such as azathioprine, cyclophosphamide or mofetil) |
| 5 | 🞎 | Cyclosporine (such as cyclosporine A or cytoxin) |
| 6 | 🞎 | Intravenous Immunoglobulins (IVIg) |
| 7 | 🞎 | Platelet transfusion |
| 8 | 🞎 | Rho(D) immune globulin (Anti-D) |
| 9 | 🞎 | Corticosteroids / steroids (such as prednisolone, methylprednisolone or dexamethasone) |
| 10 | 🞎 | Thrombopoietin receptor agonists (such as romiplostim or eltrombopag) |
| 11 | 🞎 | Other |
| 12 | 🞎 | Can’t remember the name of treatment |
| 13 | ⭘ | No therapy |

Q7. How long have you been receiving your most recently initiated treatment? <DO NOT ASK IF EQ6a OR b = code13>

Please enter a number and select the relevant unit below

| _________ | ⭘ Weeks | ⭘ Months | ⭘ Years | ⭘ Don’t know |
| --- | --- | --- | --- | --- |

| Range | Weeks : 0-52, Months: 0-12, Years: 0-10 |
| --- | --- |

Q8. Have you experienced any of the following as a consequence of receiving [INSERT RESPECTIVE TREATMENT CLASS FROM EQ6b] for the treatment of ITP? <REPEAT THE ABOVE QUESTION TEXT FOR Q8a-d IF CODE SELECTED AT EQ6b>

Please select all those you have experienced either during the treatment or afterwards

1. Anti-CD20 (such as rituximab, veltuzumab or ofatumumab) ASK IF EQ6b = code 2>
2. Corticosteroids / steroids(such as prednisolone, methylprednisolone or dexamethasone) <ASK IF EQ6b =code 10>
3. Intravenous Immunoglobins (IVIg <ASK IF EQ6b = code 6>
4. Thrombopoietin receptor agonists (such as romiplostim or eltrombopag) <ASK IF EQ6b = code 11>

<SHOW EACH ELIGIBLE COLUMN ON A SEPARATE PAGE IN THE SURVEY>

|  |  | a) | b) | c) | d) |
| --- | --- | --- | --- | --- | --- |
| 1 | Abnormal hunger | 🞎 | 🞎 | 🞎 | 🞎 |
| 2 | Acne | 🞎 | 🞎 | 🞎 | 🞎 |
| 3 | Anger and/or irritability | 🞎 | 🞎 | 🞎 | 🞎 |
| 4 | Anxiety and/or nervousness | 🞎 | 🞎 | 🞎 | 🞎 |
| 5 | Back aches and pains | 🞎 | 🞎 | 🞎 | 🞎 |
| 6 | Body aches and pains (joint stiffness, muscle cramps) | 🞎 | 🞎 | 🞎 | 🞎 |
| 7 | Bruising around injection site | 🞎 | 🞎 | 🞎 | 🞎 |
| 8 | Changes in face shape, bloating, swelling | 🞎 | 🞎 | 🞎 | 🞎 |
| 9 | Change in taste in my mouth | 🞎 | 🞎 | 🞎 | 🞎 |
| 10 | Chills | 🞎 | 🞎 | 🞎 | 🞎 |
| 11 | Cough | 🞎 | 🞎 | 🞎 | 🞎 |
| 12 | Depression | 🞎 | 🞎 | 🞎 | 🞎 |
| 13 | Diarrhea | 🞎 | 🞎 | 🞎 | 🞎 |
| 14 | Difficulty sleeping | 🞎 | 🞎 | 🞎 | 🞎 |
| 15 | Dizziness | 🞎 | 🞎 | 🞎 | 🞎 |
| 16 | Fatigue | 🞎 | 🞎 | 🞎 | 🞎 |
| 17 | Fever | 🞎 | 🞎 | 🞎 | 🞎 |
| 18 | Generalized weakness | 🞎 | 🞎 | 🞎 | 🞎 |
| 19 | Hair loss | 🞎 | 🞎 | 🞎 | 🞎 |
| 20 | Headaches | 🞎 | 🞎 | 🞎 | 🞎 |
| 21 | Heartburn | 🞎 | 🞎 | 🞎 | 🞎 |
| 22 | Heavy menstrual bleeding | 🞎 | 🞎 | 🞎 | 🞎 |
| 23 | High blood pressure | 🞎 | 🞎 | 🞎 | 🞎 |
| 24 | Hirsutism (increased hair on face or body | 🞎 | 🞎 | 🞎 | 🞎 |
| 25 | Hot flushes and/or sweating | 🞎 | 🞎 | 🞎 | 🞎 |
| 26 | Impaired wound healing | 🞎 | 🞎 | 🞎 | 🞎 |
| 27 | Increased infections, either frequency of or length of time they last | 🞎 | 🞎 | 🞎 | 🞎 |
| 28 | Increased thirst or urination | 🞎 | 🞎 | 🞎 | 🞎 |
| 29 | Infusion reactions during the infusion such as fever, chills, abdominal pain | 🞎 | 🞎 | 🞎 | 🞎 |
| 30 | Insomnia, restlessness and/or trouble sleeping | 🞎 | 🞎 | 🞎 | 🞎 |
| 31 | Muscle weakness | 🞎 | 🞎 | 🞎 | 🞎 |
| 32 | Nasopharyngitis (inflammation of the nasal cavities and/or throat) | 🞎 | 🞎 | 🞎 | 🞎 |
| 33 | Nausea, upset stomach, vomiting, | 🞎 | 🞎 | 🞎 | 🞎 |
| 34 | Night sweats | 🞎 | 🞎 | 🞎 | 🞎 |
| 35 | Osteoporosis (reduction in bone density) | 🞎 | 🞎 | 🞎 | 🞎 |
| 36 | Kidney disease | 🞎 | 🞎 | 🞎 | 🞎 |
| 37 | Respiratory symptoms (e.g. breathlessness) | 🞎 | 🞎 | 🞎 | 🞎 |
| 38 | Skin rash | 🞎 | 🞎 | 🞎 | 🞎 |
| 39 | Skin thinning | 🞎 | 🞎 | 🞎 | 🞎 |
| 40 | Stretch marks | 🞎 | 🞎 | 🞎 | 🞎 |
| 41 | Swelling of the hands or feet | 🞎 | 🞎 | 🞎 | 🞎 |
| 42 | Thrombosis (clotting too much) | 🞎 | 🞎 | 🞎 | 🞎 |
| 43 | Tingling of the hands or feet | 🞎 | 🞎 | 🞎 | 🞎 |
| 44 | Trouble with blood glucose levels, diabetes | 🞎 | 🞎 | 🞎 | 🞎 |
| 45 | Vision problems (light sensitivity/decreased ability to see or read/cataracts/increased eye pressure (glaucoma) | 🞎 | 🞎 | 🞎 | 🞎 |
| 46 | Weight gain / increased appetite | 🞎 | 🞎 | 🞎 | 🞎 |
| 47 | Weight loss | 🞎 | 🞎 | 🞎 | 🞎 |
| 48 | None | ⭘ | ⭘ | ⭘ | ⭘ |

Q9. How much do you agree with the following statements in relation to your anti-CD20 treatment (such as rituximab, veultuzumab or ofatumumab) for your ITP condition? <ASK IF EQ6b = code 2>

Please rate each statement below, where 1 (strongly disagree); to 7 (strongly agree) or D/K if you don’t know or unsure

|  |  | 1 | 2 | 3 | 4 | 5 | 6 | 7 | D/K |
| --- | --- | --- | --- | --- | --- | --- | --- | --- | --- |
| 1 | Overall, I am very satisfied with my anti-CD20 treatment’s control of my ITP condition | ⭘ | ⭘ | ⭘ | ⭘ | ⭘ | ⭘ | ⭘ | ⭘ |
| 2 | Anti-CD20 treatment is effective at treating my ITP symptoms | ⭘ | ⭘ | ⭘ | ⭘ | ⭘ | ⭘ | ⭘ | ⭘ |
| 3 | Anti-CD20 treatment increases my energy levels | ⭘ | ⭘ | ⭘ | ⭘ | ⭘ | ⭘ | ⭘ | ⭘ |
| 4 | I am worried about the short-term physical side effects of anti-CD20 treatment (e.g. headache, fever, nausea, etc.) | ⭘ | ⭘ | ⭘ | ⭘ | ⭘ | ⭘ | ⭘ | ⭘ |
| 5 | I worry about the long term side effects of anti-CD20s (e.g. increased infections ) | ⭘ | ⭘ | ⭘ | ⭘ | ⭘ | ⭘ | ⭘ | ⭘ |
| 6 | I would recommend anti-CD20 treatment to another ITP patient | ⭘ | ⭘ | ⭘ | ⭘ | ⭘ | ⭘ | ⭘ | ⭘ |
| 7 | I am worried about the immunosuppressive effect of anti-CD20 treatment | ⭘ | ⭘ | ⭘ | ⭘ | ⭘ | ⭘ | ⭘ | ⭘ |
| 8 | I do not want to take anti-CD20 treatment for the foreseeable future | ⭘ | ⭘ | ⭘ | ⭘ | ⭘ | ⭘ | ⭘ | ⭘ |
| 9 | My anti-CD20 treatment has been effective at preventing bleeding events | ⭘ | ⭘ | ⭘ | ⭘ | ⭘ | ⭘ | ⭘ | ⭘ |
| 10 | I am concerned about how much time I have to take off work for in-hospital administration of anti-CD20 treatment | ⭘ | ⭘ | ⭘ | ⭘ | ⭘ | ⭘ | ⭘ | ⭘ |

Q10. How much do you agree with the following statements in relation to your corticosteroid /steroid treatment (such as prednisolone, methylprednisolone or dexamethasone) for your ITP condition? <ASK IF EQ6b = code 10>

Please rate each statement below, where 1 (strongly disagree); to 7 (strongly agree) or D/K if you don’t know or unsure

|  |  | 1 | 2 | 3 | 4 | 5 | 6 | 7 | D/K |
| --- | --- | --- | --- | --- | --- | --- | --- | --- | --- |
| 1 | Overall, I am very satisfied with my corticosteroid/steroid treatment’s control of my ITP condition | ⭘ | ⭘ | ⭘ | ⭘ | ⭘ | ⭘ | ⭘ | ⭘ |
| 2 | Corticosteroids / steroids are effective at treating my ITP symptoms | ⭘ | ⭘ | ⭘ | ⭘ | ⭘ | ⭘ | ⭘ | ⭘ |
| 3 | Over the long term, corticosteroids/steroids increase my energy levels | ⭘ | ⭘ | ⭘ | ⭘ | ⭘ | ⭘ | ⭘ | ⭘ |
| 4 | I am worried about the short-term physical side effects of corticosteroids / steroids (bloating, weight gain, raised blood pressure, acne) | ⭘ | ⭘ | ⭘ | ⭘ | ⭘ | ⭘ | ⭘ | ⭘ |
| 5 | I am worried about the longer term physical side effects of corticosteroids / steroids (e.g. stretch marks, osteoporosis, diabetes, high bloody pressure, skin thinning, poor wound healing) | ⭘ | ⭘ | ⭘ | ⭘ | ⭘ | ⭘ | ⭘ | ⭘ |
| 6 | I would recommend corticosteroid/steroids to another ITP patient | ⭘ | ⭘ | ⭘ | ⭘ | ⭘ | ⭘ | ⭘ | ⭘ |
| 7 | I am worried about the short-term emotional side effects of corticosteroids / steroids (depression, anxiety, etc.) | ⭘ | ⭘ | ⭘ | ⭘ | ⭘ | ⭘ | ⭘ | ⭘ |
| 8 | I am worried about the immunosuppressive effect of corticosteroids / steroids | ⭘ | ⭘ | ⭘ | ⭘ | ⭘ | ⭘ | ⭘ | ⭘ |
| 9 | My corticosteroids / steroid treatment has been effective at preventing bleeding events | ⭘ | ⭘ | ⭘ | ⭘ | ⭘ | ⭘ | ⭘ | ⭘ |
| 10 | I do not want to take corticosteroids for the foreseeable future | ⭘ | ⭘ | ⭘ | ⭘ | ⭘ | ⭘ | ⭘ | ⭘ |

Q11a. How much do you agree with the following statements in relation to your thrombopoietin receptor agonist treatment (such as romiplostim or eltrombopag) for your ITP condition? <ASK IF EQ6b = code 11>

Please rate each statement below, where 1 (strongly disagree); to 7 (strongly agree) or D/K if you don’t know or unsure

|  |  | 1 | 2 | 3 | 4 | 5 | 6 | 7 | D/K |
| --- | --- | --- | --- | --- | --- | --- | --- | --- | --- |
| 1 | Overall, I am very satisfied with my Thrombopoietin receptor agonist treatment’s control of my ITP | ⭘ | ⭘ | ⭘ | ⭘ | ⭘ | ⭘ | ⭘ | ⭘ |
| 2 | Thrombopoietin receptor agonist treatment has been effective in treating my ITP symptoms | ⭘ | ⭘ | ⭘ | ⭘ | ⭘ | ⭘ | ⭘ | ⭘ |
| 3 | Thrombopoietin receptor agonist treatment increases my energy levels | ⭘ | ⭘ | ⭘ | ⭘ | ⭘ | ⭘ | ⭘ | ⭘ |
| 4 | I worry about the short term side effects of thrombopoietin receptor agonists | ⭘ | ⭘ | ⭘ | ⭘ | ⭘ | ⭘ | ⭘ | ⭘ |
| 5 | I worry about the long term side effects of thrombopoietin receptor agonists | ⭘ | ⭘ | ⭘ | ⭘ | ⭘ | ⭘ | ⭘ | ⭘ |
| 6 | I would recommend thrombopoietin receptor agonists to anther ITP patient | ⭘ | ⭘ | ⭘ | ⭘ | ⭘ | ⭘ | ⭘ | ⭘ |
| 7 | I value that thrombopoietin receptor agonists do not suppress my immune system | ⭘ | ⭘ | ⭘ | ⭘ | ⭘ | ⭘ | ⭘ | ⭘ |
| 8 | My doctor discussed the chances of sustained remission prior to beginning my treatment with thrombopoietin receptor agonists | ⭘ | ⭘ | ⭘ | ⭘ | ⭘ | ⭘ | ⭘ | ⭘ |
| 9 | My thrombopoietin receptor agonist treatment has been effective at preventing bleeding events | ⭘ | ⭘ | ⭘ | ⭘ | ⭘ | ⭘ | ⭘ | ⭘ |
| 10 | I do not want to take thrombopoietin receptor agonists for the foreseeable future | ⭘ | ⭘ | ⭘ | ⭘ | ⭘ | ⭘ | ⭘ | ⭘ |

Q11b. Was a thrombopoietin receptor agonist the last treatment you received? <ASK IF EQ6b = code 11>

| ⭘ | Yes |
| --- | --- |
| ⭘ | No |
| ⭘ | Don’t know |

Q11c. Are you in a period of sustained remission (i.e. the severity of your disease has subsided) in your ITP? <ASK IF EQ11b = code 1>

| ⭘ | Yes |
| --- | --- |
| ⭘ | No |
| ⭘ | Don’t know |

Q11d. How long have you been in clinical remission (i.e. no bleeding and no treatment)for? <ASK IF EQ11c = code 1>

| ⭘ | 0-3 months |
| --- | --- |
| ⭘ | 4-6 months |
| ⭘ | 7-12 months |
| ⭘ | >12 months |

Q12. How much do you agree with the following statements in relation to your Intravenous Immunoglobin (IVIg) treatment for your ITP condition? <ASK IF EQ6b = code 6>

Please rate each statement below, where 1 (strongly disagree); to 7 (strongly agree) or D/K if you don’t know or unsure

|  |  | 1 | 2 | 3 | 4 | 5 | 6 | 7 | D/K |
| --- | --- | --- | --- | --- | --- | --- | --- | --- | --- |
| 1 | Overall, I am very satisfied with my intravenous immunoglobulin treatment’s control of my ITP | ⭘ | ⭘ | ⭘ | ⭘ | ⭘ | ⭘ | ⭘ | ⭘ |
| 2 | Intravenous immunoglobin treatment has been effective in treating my ITP symptoms | ⭘ | ⭘ | ⭘ | ⭘ | ⭘ | ⭘ | ⭘ | ⭘ |
| 3 | Intravenous immunoglobin treatment increases my energy levels | ⭘ | ⭘ | ⭘ | ⭘ | ⭘ | ⭘ | ⭘ | ⭘ |
| 4 | I am worried about the short term side effects of intravenous immunoglobin treatment | ⭘ | ⭘ | ⭘ | ⭘ | ⭘ | ⭘ | ⭘ | ⭘ |
| 5 | I am worried about the long term side effects of intravenous immunoglobin treatment | ⭘ | ⭘ | ⭘ | ⭘ | ⭘ | ⭘ | ⭘ | ⭘ |
| 6 | I would recommend intravenous immunoglobin treatment to another ITP patient | ⭘ | ⭘ | ⭘ | ⭘ | ⭘ | ⭘ | ⭘ | ⭘ |
| 7 | I am worried about the immunosuppressive effect of intravenous immunoglobin treatment | ⭘ | ⭘ | ⭘ | ⭘ | ⭘ | ⭘ | ⭘ | ⭘ |
| 8 | I find intravenous immunoglobin treatment administration to be inconvenient | ⭘ | ⭘ | ⭘ | ⭘ | ⭘ | ⭘ | ⭘ | ⭘ |
| 9 | I do not want to take intravenous immunoglobin treatment for the foreseeable future | ⭘ | ⭘ | ⭘ | ⭘ | ⭘ | ⭘ | ⭘ | ⭘ |
| 10 | I am concerned about how much time I have to take off work for in-hospital administration of intravenous immunoglobins | ⭘ | ⭘ | ⭘ | ⭘ | ⭘ | ⭘ | ⭘ | ⭘ |
| 11 | I find it burdensome attending the hospital so frequently for intravenous immunoglobin treatment | ⭘ | ⭘ | ⭘ | ⭘ | ⭘ | ⭘ | ⭘ | ⭘ |

Q13. How important is it to you that the treatments you receive to manage your ITP are licensed? <ASK ALL>

*Licensed therapies undergo rigorous clinical trials examining safety and efficacy and are registered with Health Authorities such as the European Medicines Agency and the Food and Drug Administration.*

Please select a rating below, where 1 (not at all); to 7 (very much so)

| 1  ⭘ | 2  ⭘ | 3  ⭘ | 4  ⭘ | 5  ⭘ | 6  ⭘ | 7  ⭘ |
| --- | --- | --- | --- | --- | --- | --- |

Q14a. Other than a cure for your ITP condition, what are your 3 most important treatment goals? <ASK ALL>

Please assign rankings (1 to 3) with 1 being the most important

| 1 | 🞎 | Increasing my energy levels |
| --- | --- | --- |
| 2 | 🞎 | Reducing my spontaneous bleeds/bruising |
| 3 | 🞎 | Healthy blood counts (i.e. stable good platelet count) |
| 4 | 🞎 | Preventing episodes of worsening of my ITP |
| 5 | 🞎 | Improving my quality of life |
| 6 | 🞎 | Improvement in overall symptoms |
| 7 | 🞎 | Lighter menstrual periods <Only if AQ1 = code 2> |
| 8 | 🞎 | Reducing general weakness |
| 9 | 🞎 | Preventing the development of blood clots (even if very rare) |
| 10 | 🞎 | Improvement in emotional mood (less anxiety, depressed feelings) |

Q14b. Do you feel that your current treatment approach is helping you towards reaching these treatment goals at all? <ASK ALL>

Please select one answer

| ⭘ | Strongly agree |
| --- | --- |
| ⭘ | Somewhat agree |
| ⭘ | Somewhat disagree |
| ⭘ | Strongly disagree |
| ⭘ | No opinion / don’t know |

Q14c. Is your current treating doctor completely aware of your treatment goals? <ASK ALL>

Please select one answer

| ⭘ | Yes |
| --- | --- |
| ⭘ | No |
| ⭘ | Don’t know |

Q15a. How stable are your platelet counts currently? <ASK ALL>

Please select one answer

| ⭘ | Not currently stable |
| --- | --- |
| ⭘ | They have been stable for the last month |
| ⭘ | They have been stable for the last 2-6 months |
| ⭘ | They have been stable for the last 7-12 months |
| ⭘ | They have been stable for over 1 year |
| ⭘ | Don’t know |

Q15b. What was your last platelet count? <ASK ALL>

Please select one answer

| 1 | ⭘ <10 (x10^9^/L) <US and JP only (x10^3^/µL)> |
| --- | --- |
| 2 | ⭘ 10-29 (x10^9^/L) <US and JP only (x10^3^/µL)> |
| 3 | ⭘ 30-39 (x10^9^/L) <US and JP only (x10^3^/µL)> |
| 4 | ⭘ 40-49 (x10^9^/L) <US and JP only (x10^3^/µL)> |
| 5 | ⭘ 50-69 (x10^9^/L) <US and JP only (x10^3^/µL)> |
| 6 | ⭘ 70-100 (x10^9^/L) <US and JP only (x10^3^/µL)> |
| 7 | ⭘ > 100 (x10^9^/L) <US and JP only (x10^3^/µL)> |
| 8 | ⭘ Don’t know |

Q16. How important do you consider the following aspects when making treatment decisions to manage your ITP condition? <ASK ALL>

**Please indicate on a scale of 1 to 100, where 1 = not important at all and 100 = of the highest importance**

| a | Prevention of immunosuppression | 100 point sliding scale |
| --- | --- | --- |
| b | Reduction of bleeding risk | 100 point sliding scale |
| c | Quick recovery but with a risk of recovery being temporary | 100 point sliding scale |
| d | Slower recovery from relapse, but potentially longer term | 100 point sliding scale |
| e | Keeping side effects to a minimum | 100 point sliding scale |
| f | Ability to offer sustained remission or cure of ITP | 100 point sliding scale |

Q17. Please select your preference in terms of how you would like to take your ITP medicine if you had a choice

Please select one answer

| ⭘ | Once daily oral pill |
| --- | --- |
| ⭘ | Twice daily oral pill |
| ⭘ | Needle / injection |

END OF SECTION

SECTION F: PATIENT AND PHYSICIAN RELATIONSHIP

Q1a. Which health care professionals have you seen in the past 12 months to manage your ITP? <ASK ALL>

Q1b. How many times have you seen this person in the past 12 months about your ITP? <ONLY SHOW WHERE ANSWER IS SELECTED AT FQ1a>

Q1c. How do you feel the frequency you are seeing this person to be: <ONLY SHOW WHERE ANSWER IS SELECTED AT FQ1a>

Please select all that apply

|  | a) | b) | c) |
| --- | --- | --- | --- |
| 🞎 | GP/Family Doctor | _____ | ⭘ Not enough  ⭘ Enough  ⭘ Too much |
| 🞎 | Specialist Doctor | _____ | ⭘ Not enough  ⭘ Enough  ⭘ Too much |
| 🞎 | Specialist Nurse | _____ | ⭘ Not enough  ⭘ Enough  ⭘ Too much |
| 🞎 | Psychologist | _____ | ⭘ Not enough  ⭘ Enough  ⭘ Too much |
| 🞎 | Other HCP |  |  |

Q2. Who do you consider to be the main person responsible for the treatment and management of your ITP? <ONLY SHOW OPTIONS IF SELCETED AT Q1a, IF ONE OPTION SELECTED AT Q1a THEN SKIP>

| ⭘ | GP/Family Doctor |
| --- | --- |
| ⭘ | Specialist Doctor (such as a hematologist) |
| ⭘ | Specialist Nurse |
| ⭘ | Other HCP |

Q3. Thinking about your <INSERT ANSWER FROM FQ2> who has responsibility for your ITP treatment and management, overall how satisfied are you with their…<ASK ALL>

Please rate each statement below, where 1 (very dissatisfied); to 7 (very satisfied).

|  |  | 1  Very dissatisfied | 2 | 3 | 4 | 5 | 6 | 7  Very satisfied |
| --- | --- | --- | --- | --- | --- | --- | --- | --- |
| a. | **Communication** about your ITP condition and its treatment | ⭘ | ⭘ | ⭘ | ⭘ | ⭘ | ⭘ | ⭘ |
| b. | **Management** **and treatment** of your ITP condition | ⭘ | ⭘ | ⭘ | ⭘ | ⭘ | ⭘ | ⭘ |
| c. | **Understanding and support** of your treatment goals | ⭘ | ⭘ | ⭘ | ⭘ | ⭘ | ⭘ | ⭘ |
| d. | **Consideration of your needs** in designing the treatment goals | ⭘ | ⭘ | ⭘ | ⭘ | ⭘ | ⭘ | ⭘ |

Q4. Which statement best describes how your <INSERT ANSWER FROM FQ2> explained the potential ITP symptoms you may experience over the course of the disease, if they did at all? <ASK ALL>

Please select one answer

| ⭘ | They ran through a full and comprehensive list of symptoms |
| --- | --- |
| ⭘ | They outlined symptoms I may experience |
| ⭘ | They mentioned the two or three most bothersome symptoms I may experience |
| ⭘ | They didn’t discuss anything beyond symptoms I already have |
| ⭘ | They referred me to a nurse or other doctor to have a more in-depth discussion about my symptoms |
| ⭘ | There was no discussion about my symptoms (either currently experiencing or could potentially experience), only about which treatment to use |
| ⭘ | Other |

Q5. Based on your experience with your <INSERT ANSWER FROM FQ2>, to what extent do you agree with the following statements with regards to your ITP condition? <<ASK ALL>

Please rate each statement below, where 1 (strongly disagree); to 7 (strongly agree).

|  |  | 1 Strongly disagree | 2 | 3 | 4 | 5 | 6 | 7 Strongly agree |
| --- | --- | --- | --- | --- | --- | --- | --- | --- |
| 1 | My <FQ2> is genuinely concerned about helping me | ⭘ | ⭘ | ⭘ | ⭘ | ⭘ | ⭘ | ⭘ |
| 2 | My <FQ2> listens to my concerns and addresses my questions | ⭘ | ⭘ | ⭘ | ⭘ | ⭘ | ⭘ | ⭘ |
| 3 | My <FQ2> is aware of my concerns around immunosuppression | ⭘ | ⭘ | ⭘ | ⭘ | ⭘ | ⭘ | ⭘ |
| 4 | My <FQ2> keeps me informed about new treatment options | ⭘ | ⭘ | ⭘ | ⭘ | ⭘ | ⭘ | ⭘ |
| 5 | My <FQ2> has created a treatment plan for therapy that we have discussed together | ⭘ | ⭘ | ⭘ | ⭘ | ⭘ | ⭘ | ⭘ |
| 6 | My <FQ2> asks me about my symptoms at every appointment | ⭘ | ⭘ | ⭘ | ⭘ | ⭘ | ⭘ | ⭘ |
| 7 | My <FQ2> understands how much my condition impacts my life | ⭘ | ⭘ | ⭘ | ⭘ | ⭘ | ⭘ | ⭘ |
| 8 | I feel comfortable discussing my symptoms with my <FQ2> | ⭘ | ⭘ | ⭘ | ⭘ | ⭘ | ⭘ | ⭘ |
| 9 | There is always time during the consultation to discuss all my symptoms and even other problems | ⭘ | ⭘ | ⭘ | ⭘ | ⭘ | ⭘ | ⭘ |
| 10 | My <FQ2> explains my test results to me and how they could be causing or explaining my symptoms | ⭘ | ⭘ | ⭘ | ⭘ | ⭘ | ⭘ | ⭘ |
| 11 | I am able to accurately describe my symptoms to my <FQ2> | ⭘ | ⭘ | ⭘ | ⭘ | ⭘ | ⭘ | ⭘ |
| 12 | I am confident that I am being assessed and treated properly by my <FQ2> | ⭘ | ⭘ | ⭘ | ⭘ | ⭘ | ⭘ | ⭘ |
| 13 | My <FQ2> and I have similar goals for my ITP management and/or treatment | ⭘ | ⭘ | ⭘ | ⭘ | ⭘ | ⭘ | ⭘ |
| 14 | My <FQ2> explained the potential long-term complications of a splenectomy to me before I had the procedure <ONLY ASK IF SELECTED EQ5 code 1> | ⭘ | ⭘ | ⭘ | ⭘ | ⭘ | ⭘ | ⭘ |
| 15 | My <FQ2> explains the potential drug side effects before giving me a treatment | ⭘ | ⭘ | ⭘ | ⭘ | ⭘ | ⭘ | ⭘ |
| 16 | My <FQ2> is aware of my concerns about my treatments | ⭘ | ⭘ | ⭘ | ⭘ | ⭘ | ⭘ | ⭘ |
| 17 | My <FQ2> is up to date with the best current treatments and advances in ITP care | ⭘ | ⭘ | ⭘ | ⭘ | ⭘ | ⭘ | ⭘ |

Q6a. Did your <INSERT ANSWER FROM FQ2> give you any other information (e.g. leaflets, websites, etc.) to further explain the potential ITP symptoms you may experience? <ASK ALL>

Please select one answer

| ⭘ | Yes |
| --- | --- |
| ⭘ | No <Skip to FQ9> |

Q6b. What kind of information were you given?

Please select one answer

| ⭘ | My <FQ2> provided me with a leaflet and explained the content for me |
| --- | --- |
| ⭘ | My <FQ2> provided me with a leaflet with no explanation |
| ⭘ | My <FQ2> brought up a website during the consultation and explained the content for me – I was able to go away and research my disease alone |
| ⭘ | My <FQ2> directed me to a website(s) with more information on my disease |
| ⭘ | My <FQ2> told me about Patient Support Groups in ITP to find out more about my disease |
| ⭘ | My <FQ2> went through ITP disease information with me using leaflets, websites, etc. |

Q7. Are you in contact with a patient support group or patient organization for ITP? <ASK ALL>

Please select one answer

| ⭘ | Yes |
| --- | --- |
| ⭘ | No |

END OF SURVEY
