# Supplementary material for: Immune thrombocytopenia (ITP) World Impact Survey (I‐WISh): Impact of ITP on health‐related quality of life
Source: Am J Hematol. 2020 Dec 19;96(2):199–207. doi: 10.1002/ajh.26036 (PMC7898815; doi:10.1002/ajh.26036)
Supplement: Supplementary file 3 — Appendix S3. Supporting Information. [file AJH-96-199-s003.docx]

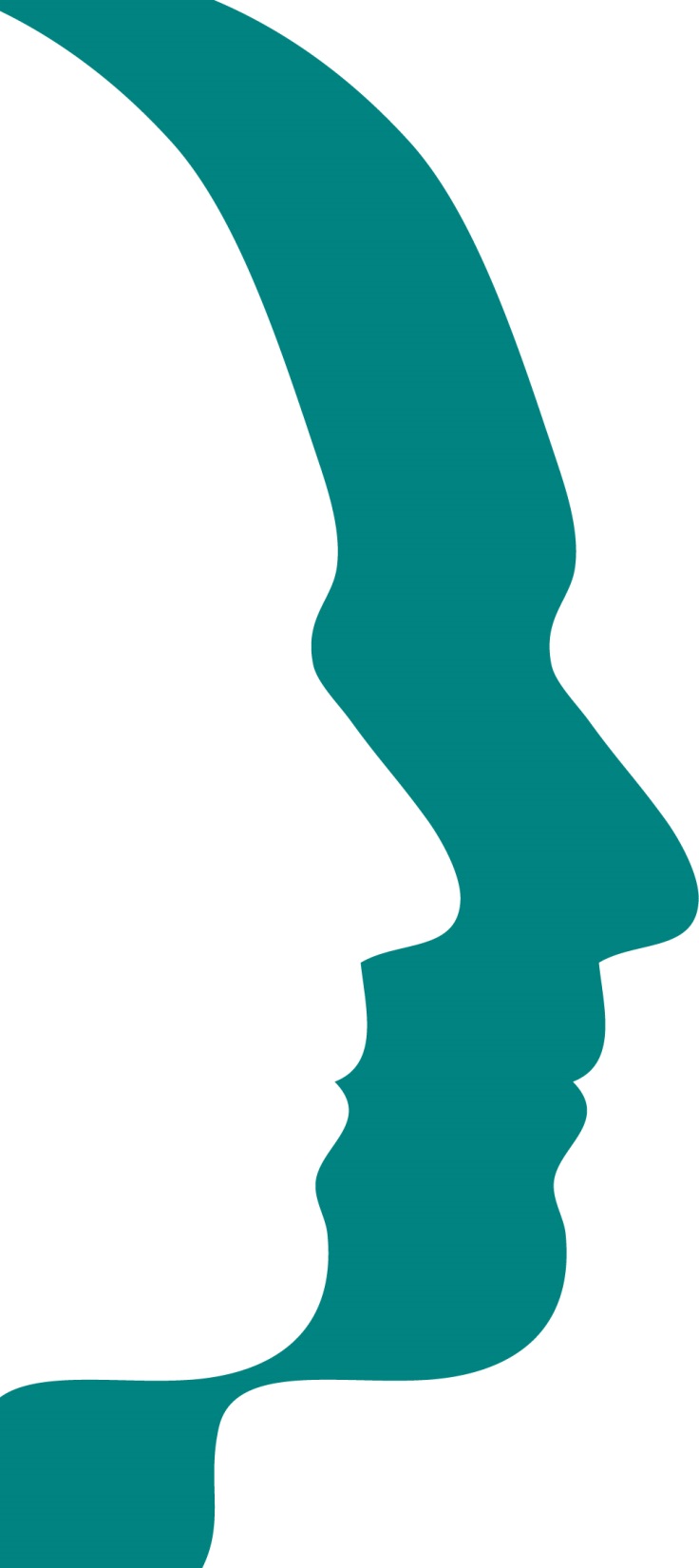

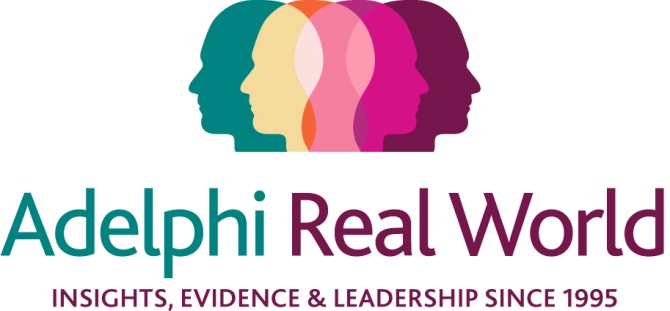


**NO 8348 ITP**

**PHYSICIAN SURVEY**

V11.0

December 2017

PHYSICIAN SCREENER

S1. What is your primary speciality? <ASK ALL>

Please select one answer

| ⭘ | Haematology |
| --- | --- |
| ⭘ | Haematology-Oncology |
| ⭘ | Other |

S2. Please answer the below questions about your patient caseload: <ASK ALL>

Please enter an estimated number into each column

|  | a. Your total patient caseload | b. Number of ITP patients currently under your care | c. Number of ITP patients under your care in past 12 months | d. Newly diagnosed ITP (under your care) in past 12 months |
| --- | --- | --- | --- | --- |
| Number of patients: | ________ | ________ | ________ | ________ |

S3. Are you responsible for making treatment decisions for the ITP patients under your care? <ASK ALL>

Please select one answer

| ⭘ | Yes |
| --- | --- |
| ⭘ | No |

SECTION A: PHYSICIAN DEMOGRAPHICS

Q1. In what year did you qualify as a <If S1 = Hematology then insert Hematologist, If S1 = Hematology-Oncology then insert Hematologist-Oncologist>? <ASK ALL>

Please select one answer

| ⭘ | Before 1981 |
| --- | --- |
| ⭘ | 1981-1993 |
| ⭘ | 1994-2003 |
| ⭘ | 2004-2014 |
| ⭘ | After 2014 |

Q2. In what type of care setting do you spend most of your patient care time? <ASK ALL>

Please select one answer

| ⭘ | Specialist cancer centre |
| --- | --- |
| ⭘ | University/Teaching hospital |
| ⭘ | Regional/Community hospital |
| ⭘ | Private hospital |
| ⭘ | Office based <DE only> |
| ⭘ | Other |

SECTION B: DIAGNOSIS OF ITP AND CASELOAD

Q1. Of your total patient case load, where do you prioritise ITP cases? <ASK ALL>

**Please select the most appropriate response**

| ⭘ | I consider my ITP case load my **highest priority** |
| --- | --- |
| ⭘ | I consider my ITP case load **somewhat less important** than other more serious conditions |
| ⭘ | I consider my ITP case load **significantly less important** than other more serious conditions |

Q2. Considering all your ITP patients, what proportion do you estimate have: <ASK ALL>

**Please add a percentage for each response ensuring your answers total 100%**

| Primary ITP | ________ % |
| --- | --- |
| Secondary ITP | ________ % |
| **TOTAL** <MUST EQUAL 100%> | **100%** |

Q3. In your experience, which three conditions are the most common cause of secondary ITP? <ASK ALL>

**Please select the 3 conditions, starting with the most common**

| 1 | Antiphospholipid syndrome (APS) | 🞎 |
| --- | --- | --- |
| 2 | Autoimmune lymphoproliferative syndrome (post-treatment) | 🞎 |
| 3 | CD8 T-lymphocyte large granular lymphocytic leukaemia (LGL) | 🞎 |
| 4 | Chronic lymphocytic leukaemia (CLL) | 🞎 |
| 5 | Common variable immunodeficiency (CVID) | 🞎 |
| 6 | Drug-induced thrombocytopenia (DITP), e.g. after receiving heparin, gold salts and antibiotics. | 🞎 |
| 7 | Evans disease/syndrome (ES) | 🞎 |
| 8 | H pylori infection | 🞎 |
| 9 | Hepatitis C virus | 🞎 |
| 10 | Hodgkin’s Lymphoma | 🞎 |
| 11 | Human immunodeficiency virus (HIV) | 🞎 |
| 12 | Systemic lupus erythematosus (SLE) | 🞎 |
| 13 | Thyroid disease | 🞎 |
| 14 | Post vaccination | 🞎 |
| 15 | Other condition | 🞎 |

Q4. On average, how long do you estimate it takes to formally diagnose a patient with ITP following their initial symptom presentation to a doctor? <ASK ALL>

Please consider any misdiagnoses the patient may have received and the time a patient may have spent in another health care setting or with another physician as part of the overall diagnosis process.

| _________ | ⭘ Weeks | ⭘ Months | ⭘ Years | ⭘ Don’t know |
| --- | --- | --- | --- | --- |

Q5. Considering your own experience, what impact does the following have on the potential to delay the formal diagnosis of ITP? <ASK ALL>

Please rate the following options, where 1 (no impact); to 7 (very high impact)

|  | 1 | 2 | 3 | 4 | 5 | 6 | 7 |
| --- | --- | --- | --- | --- | --- | --- | --- |
| Misdiagnosis of ITP as other condition | ⭘ | ⭘ | ⭘ | ⭘ | ⭘ | ⭘ | ⭘ |
| Exclusion of other potential causes | ⭘ | ⭘ | ⭘ | ⭘ | ⭘ | ⭘ | ⭘ |
| Time taken to refer the patient to the relevant specialist | ⭘ | ⭘ | ⭘ | ⭘ | ⭘ | ⭘ | ⭘ |
| Diagnostic examination (including medical history, physical examination, blood counts etc.) | ⭘ | ⭘ | ⭘ | ⭘ | ⭘ | ⭘ | ⭘ |

Q6a. In your experience, how frequently are patients misdiagnosed? <ASK ALL>

| ⭘ | Never |
| --- | --- |
| ⭘ | 1-25% of the time |
| ⭘ | 26 – 50% of the time |
| ⭘ | 51 – 75% of the time |
| ⭘ | 76 – 100% of the time |

Q6b. In your experience, what are the 3 most common conditions patients are misdiagnosed with prior to their formal ITP diagnosis? <ASK IF Q6a IS NOT ‘NEVER’>

**Please select up to 3 conditions, starting with the most common**

| Metastatic cancer | 🞎 |
| --- | --- |
| Leukaemia | 🞎 |
| Haemophilia | 🞎 |
| Aplastic anaemia/MDS | 🞎 |
| TTP | 🞎 |
| Hereditary thrombocytopenia | 🞎 |
| Drug-induced thrombocytopenia | 🞎 |
| Liver disease | 🞎 |
| Hypersplenism | 🞎 |
| Other | 🞎 |

Q7. When ITP is suspected in the following patient groups, how likely would you be to administer the following tests? <ASK ALL>

Please select all options that apply for each patient group

|  | **Asymptomatic patients** | **Patients with low symptom burden** | **Patients with moderate symptom burden** | **Patients with high symptom burden** |
| --- | --- | --- | --- | --- |
| Peripheral blood smear | 🞎 | 🞎 | 🞎 | 🞎 |
| Bone marrow aspirate/biopsy | 🞎 | 🞎 | 🞎 | 🞎 |
| Complete blood count (CBC) | 🞎 | 🞎 | 🞎 | 🞎 |
| Computed tomography (CT) | 🞎 | 🞎 | 🞎 | 🞎 |
| Direct antiglobulin test (Coombs test) | 🞎 | 🞎 | 🞎 | 🞎 |
| HIV status | 🞎 | 🞎 | 🞎 | 🞎 |
| H. pylori testing | 🞎 | 🞎 | 🞎 | 🞎 |
| Magnetic resonance imaging (MRI) | 🞎 | 🞎 | 🞎 | 🞎 |
| Platelet specific assays (i.e. platelet antigen-specific antibodies, platelet-associated immunoglobin) | 🞎 | 🞎 | 🞎 | 🞎 |
| Spleen evaluation | 🞎 | 🞎 | 🞎 | 🞎 |
| Physical examination | 🞎 | 🞎 | 🞎 | 🞎 |
| Other | 🞎 | 🞎 | 🞎 | 🞎 |
| I do not conduct any of the above tests | **⭘** | **⭘** | **⭘** | **⭘** |

END OF SECTION

SECTION C: SYMPTOMS

Q1. What are the 5 symptoms you most frequently hear about from your ITP patients? <ASK ALL>

**Please select 5 symptoms, starting with the most frequent symptom**

| 1 | Purpura | 🞎 |
| --- | --- | --- |
| 2 | Petechiae | 🞎 |
| 3 | Haematoma |  |
| 4 | Thrombosis (arterial and/or venous) | 🞎 |
| 5 | Epistaxis | 🞎 |
| 6 | Bleeding from the gums | 🞎 |
| 7 | Prolonged bleeding from cuts | 🞎 |
| 8 | Profuse bleeding during surgery | 🞎 |
| 9 | Haematuria or melena, rectal bleeding | 🞎 |
| 10 | Menorrhagia | 🞎 |
| 11 | Fatigue | 🞎 |
| 12 | Moderate to severe headaches/ migraines | 🞎 |
| 13 | Dizziness | 🞎 |
| 14 | Depression / Psychological symptoms | 🞎 |
| 15 | Social sequela | 🞎 |
| 16 | Anxiety surrounding unstable platelet count | 🞎 |
| 17 | Other symptom | 🞎 |

Q2. In your experience, what are the 5 most common symptoms your patients present with at time of ITP diagnosis? <ASK ALL>

**Select the 5 most common symptoms, starting with the most common**

| 1 | Purpura | 🞎 |
| --- | --- | --- |
| 2 | Petechiae | 🞎 |
| 3 | Haematoma | 🞎 |
| 4 | Thrombosis (arterial and/or venous) | 🞎 |
| 5 | Epistaxis | 🞎 |
| 6 | Bleeding from the gums | 🞎 |
| 7 | Prolonged bleeding from cuts | 🞎 |
| 8 | Profuse bleeding during surgery | 🞎 |
| 9 | Haematuria or melena, rectal bleeding | 🞎 |
| 10 | Menorrhagia | 🞎 |
| 11 | Fatigue | 🞎 |
| 12 | Moderate to severe headaches / migraines | 🞎 |
| 13 | Dizziness | 🞎 |
| 14 | Depression /Psychological symptoms | 🞎 |
| 15 | Social sequela | 🞎 |
| 16 | Anxiety surrounding unstable platelet count | 🞎 |
| 17 | Other symptom | 🞎 |

Q3. In your opinion, which of the following symptoms have a major negative effect on a patient’s quality of life? <ASK ALL>

Please rate the following options, where 1 (not at all); to 7 (a great deal)

|  |  | 1 | 2 | 3 | 4 | 5 | 6 | 7 |
| --- | --- | --- | --- | --- | --- | --- | --- | --- |
| 1 | Purpura | ⭘ | ⭘ | ⭘ | ⭘ | ⭘ | ⭘ | ⭘ |
| 2 | Petechiae | ⭘ | ⭘ | ⭘ | ⭘ | ⭘ | ⭘ | ⭘ |
| 3 | Haematoma | ⭘ | ⭘ | ⭘ | ⭘ | ⭘ | ⭘ | ⭘ |
| 4 | Thrombosis (arterial and/or venous) | ⭘ | ⭘ | ⭘ | ⭘ | ⭘ | ⭘ | ⭘ |
| 5 | Epistaxis | ⭘ | ⭘ | ⭘ | ⭘ | ⭘ | ⭘ | ⭘ |
| 6 | Bleeding from the gums | ⭘ | ⭘ | ⭘ | ⭘ | ⭘ | ⭘ | ⭘ |
| 7 | Prolonged bleeding from cuts | ⭘ | ⭘ | ⭘ | ⭘ | ⭘ | ⭘ | ⭘ |
| 8 | Profuse bleeding during surgery | ⭘ | ⭘ | ⭘ | ⭘ | ⭘ | ⭘ | ⭘ |
| 9 | Haematuria or melena, rectal bleeding | ⭘ | ⭘ | ⭘ | ⭘ | ⭘ | ⭘ | ⭘ |
| 10 | Menorrhagia | ⭘ | ⭘ | ⭘ | ⭘ | ⭘ | ⭘ | ⭘ |
| 11 | Fatigue | ⭘ | ⭘ | ⭘ | ⭘ | ⭘ | ⭘ | ⭘ |
| 12 | Moderate to severe headaches / migraines | ⭘ | ⭘ | ⭘ | ⭘ | ⭘ | ⭘ | ⭘ |
| 13 | Dizziness | ⭘ | ⭘ | ⭘ | ⭘ | ⭘ | ⭘ | ⭘ |
| 14 | Depression / Psychological symptoms | ⭘ | ⭘ | ⭘ | ⭘ | ⭘ | ⭘ | ⭘ |
| 15 | Social sequela | ⭘ | ⭘ | ⭘ | ⭘ | ⭘ | ⭘ | ⭘ |
| 16 | Anxiety surrounding unstable platelet count | ⭘ | ⭘ | ⭘ | ⭘ | ⭘ | ⭘ | ⭘ |

Q4. Overall, to what extent do you feel ITP symptoms can reduce a patient’s quality of life? <ASK ALL>

Please rate the following options, where 1 (not at all); to 7 (a great deal)

|  | 1 | 2 | 3 | 4 | 5 | 6 | 7 |
| --- | --- | --- | --- | --- | --- | --- | --- |
| ITP symptoms can reduce a patient’s quality of life. | ⭘ | ⭘ | ⭘ | ⭘ | ⭘ | ⭘ | ⭘ |

Q5. Overall, to what extent do you feel ITP related fatigue can reduce a patient’s quality of life? <ASK ALL>

Please rate the following options, where 1 (not at all); to 7 (a great deal)

|  | 1 | 2 | 3 | 4 | 5 | 6 | 7 |
| --- | --- | --- | --- | --- | --- | --- | --- |
| ITP related fatigue can reduce a patient’s quality of life. | ⭘ | ⭘ | ⭘ | ⭘ | ⭘ | ⭘ | ⭘ |

SECTION D: IMPACT OF DISEASE

Q1a. What proportion of your ITP patients do you feel experience fatigue? <ASK ALL>

Please enter a percentage below

| ________ % of patients who experience fatigue |
| --- |

Q1b. Of those patients experiencing fatigue related to their ITP, please rate the severity of that fatigue? <ASK IF DQ1a>0>

Please rate patients fatigue below; where 1 (low level of fatigue) to 7 (completely fatigued)

| 1 | 2 | 3 | 4 | 5 | 6 | 7 |
| --- | --- | --- | --- | --- | --- | --- |
| ⭘  low level of fatigue | ⭘ | ⭘ | ⭘ | ⭘ | ⭘ | ⭘  completely fatigued |

Q1c. Please state how likely each of the following patient groups are to experience fatigue. <ASK ALL>

**Please rate how likely, where 1 (not at all); to 7 (very likely)**

|  |  | 1  (Not at all) | 2 | 3 | 4 | 5 | 6 | 7  (Very likely) |
| --- | --- | --- | --- | --- | --- | --- | --- | --- |
| 1 | <10 (x10^9^/L) <US and JP only (x10^3^/µL)> | **⭘** | **⭘** | **⭘** | **⭘** | **⭘** | **⭘** | **⭘** |
| 2 | 10-29 (x10^9^/L) <US and JP only (x10^3^/µL)> |  |  |  |  |  |  |  |
| 3 | 30-39 (x10^9^/L) <US and JP only (x10^3^/µL)> | **⭘** | **⭘** | **⭘** | **⭘** | **⭘** | **⭘** | **⭘** |
| 4 | 40-49 (x10^9^/L) <US and JP only (x10^3^/µL)> | **⭘** | **⭘** | **⭘** | **⭘** | **⭘** | **⭘** | **⭘** |
| 5 | 50-69 (x10^9^/L) <US and JP only (x10^3^/µL)> | **⭘** | **⭘** | **⭘** | **⭘** | **⭘** | **⭘** | **⭘** |
| 6 | 70-100 (x10^9^/L) <US and JP only (x10^3^/µL)> | **⭘** | **⭘** | **⭘** | **⭘** | **⭘** | **⭘** | **⭘** |
| 7 | > 100 (x10^9^/L) <US and JP only (x10^3^/µL)> | **⭘** | **⭘** | **⭘** | **⭘** | **⭘** | **⭘** | **⭘** |

Q2. In your opinion, do you think patients may feel/experience any of the following as a result of their ITP condition? <ASK ALL>

Please rate the following options, where 1 (not at all); to 7 (a great deal)

|  |  | 1  (not at all) | 2 | 3 | 4 | 5 | 6 | 7  (a great deal) | No opinion |
| --- | --- | --- | --- | --- | --- | --- | --- | --- | --- |
| 1 | Anxiety about platelet counts | ⭘ | ⭘ | ⭘ | ⭘ | ⭘ | ⭘ | ⭘ | ⭘ |
| 2 | Frustrations around having a long-term, rare disease | ⭘ | ⭘ | ⭘ | ⭘ | ⭘ | ⭘ | ⭘ | ⭘ |
| 3 | Depression | ⭘ | ⭘ | ⭘ | ⭘ | ⭘ | ⭘ | ⭘ | ⭘ |
| 4 | Stress | ⭘ | ⭘ | ⭘ | ⭘ | ⭘ | ⭘ | ⭘ | ⭘ |
| 5 | Feeling of helplessness | ⭘ | ⭘ | ⭘ | ⭘ | ⭘ | ⭘ | ⭘ | ⭘ |
| 6 | Worry about dying | ⭘ | ⭘ | ⭘ | ⭘ | ⭘ | ⭘ | ⭘ | ⭘ |
| 7 | Unable to express their concerns around ITP with you | ⭘ | ⭘ | ⭘ | ⭘ | ⭘ | ⭘ | ⭘ | ⭘ |
| 8 | Concerns around their physical appearance (i.e. bruising, rashes, lumps) | ⭘ | ⭘ | ⭘ | ⭘ | ⭘ | ⭘ | ⭘ | ⭘ |
| 9 | Fear around the disease (e.g. fearful of major bleeds, cerebral bleeds) | ⭘ | ⭘ | ⭘ | ⭘ | ⭘ | ⭘ | ⭘ | ⭘ |

Q3. In your opinion, to what extent do you believe ITP **negatively** affects the following aspects of patients’ lives? <ASK ALL>

Please rate the following options, where 1 (not at all); to 7 (a great deal)

|  |  | 1  (not at all) | 2 | 3 | 4 | 5 | 6 | 7  (a great deal) | No opinion |
| --- | --- | --- | --- | --- | --- | --- | --- | --- | --- |
| 1 | Daily activities (i.e. food preparation, housework, gardening, taking care of children, oral hygiene) | ⭘ | ⭘ | ⭘ | ⭘ | ⭘ | ⭘ | ⭘ | ⭘ |
| 2 | Family or social life (meeting friends/family for activities, hobbies) | ⭘ | ⭘ | ⭘ | ⭘ | ⭘ | ⭘ | ⭘ | ⭘ |
| 3 | Relationship with (informal and formal) caregiver (to those applicable) | ⭘ | ⭘ | ⭘ | ⭘ | ⭘ | ⭘ | ⭘ | ⭘ |
| 4 | Relationship with spouse/partner (to those applicable) | ⭘ | ⭘ | ⭘ | ⭘ | ⭘ | ⭘ | ⭘ | ⭘ |
| 5 | Out of pocket expenses | ⭘ | ⭘ | ⭘ | ⭘ | ⭘ | ⭘ | ⭘ | ⭘ |

Q4. In your opinion, to what extent do you believe ITP **negatively** affects patients' physical activity levels? <ASK ALL>

Please rate the following options, where 1 (not at all); to 7 (a great deal)

|  |  | 1  (not at all) | 2 | 3 | 4 | 5 | 6 | 7  (a great deal) | No opinion |
| --- | --- | --- | --- | --- | --- | --- | --- | --- | --- |
| 1 | Overall interference with level of physical activity | ⭘ | ⭘ | ⭘ | ⭘ | ⭘ | ⭘ | ⭘ | ⭘ |
| 2 | Mild physical exercise (walking, stretching) | ⭘ | ⭘ | ⭘ | ⭘ | ⭘ | ⭘ | ⭘ | ⭘ |
| 3 | Moderate physical exercise (walking up and down stairs, short walks) | ⭘ | ⭘ | ⭘ | ⭘ | ⭘ | ⭘ | ⭘ | ⭘ |
| 4 | Intense physical exercise (long fast walks/jogs, swimming, cycling, lifting weights) | ⭘ | ⭘ | ⭘ | ⭘ | ⭘ | ⭘ | ⭘ | ⭘ |
| 5 | Contact sports or sports with chance of bleeding injury (martial arts, rugby, football, tennis, etc.) | ⭘ | ⭘ | ⭘ | ⭘ | ⭘ | ⭘ | ⭘ | ⭘ |

Q5. In your opinion, to what extent do you believe ITP interferes with a patient’s sex life? <ASK ALL>

Please rate the following options, where 1 (not at all); to 7 (a great deal)

|  |  | 1  (not at all) | 2 | 3 | 4 | 5 | 6 | 7  (a great deal) | No opinion |
| --- | --- | --- | --- | --- | --- | --- | --- | --- | --- |
| 1 | Negative impact on sexual activity | ⭘ | ⭘ | ⭘ | ⭘ | ⭘ | ⭘ | ⭘ | ⭘ |
| 2 | Negative impact on sexual desire | ⭘ | ⭘ | ⭘ | ⭘ | ⭘ | ⭘ | ⭘ | ⭘ |
| 3 | Experience coital or post-coital bleeding | ⭘ | ⭘ | ⭘ | ⭘ | ⭘ | ⭘ | ⭘ | ⭘ |
| 4 | Patient concerns around post-coital bleeding | ⭘ | ⭘ | ⭘ | ⭘ | ⭘ | ⭘ | ⭘ | ⭘ |

Q6. In your opinion, how does ITP affect a patient’s travel plans? <ASK ALL>

Please rate each statement below, where 1 (not at all); to 7 (a great deal)

|  |  | 1  (not at all) | 2 | 3 | 4 | 5 | 6 | 7  (a great deal) | No opinion |
| --- | --- | --- | --- | --- | --- | --- | --- | --- | --- |
| 1 | Concerns around increased risk of bleeding | ⭘ | ⭘ | ⭘ | ⭘ | ⭘ | ⭘ | ⭘ | ⭘ |
| 2 | Burdensome for patients needing to consult about their platelet levels before travelling | ⭘ | ⭘ | ⭘ | ⭘ | ⭘ | ⭘ | ⭘ | ⭘ |
| 3 | Concerned about taking medications abroad | ⭘ | ⭘ | ⭘ | ⭘ | ⭘ | ⭘ | ⭘ | ⭘ |
| 4 | Concerns around need for travel insurance | ⭘ | ⭘ | ⭘ | ⭘ | ⭘ | ⭘ | ⭘ | ⭘ |

Q7. Do you currently use, or would you use if it was available, a patient self-completed questionnaire to assess patient quality of life (QoL) in routine clinical practice? <ASK ALL>

Please select one answer

| ⭘ | Yes - I currently use a questionnaire like this in practice |
| --- | --- |
| ⭘ | No - I do not use, but I would like to |
| ⭘ | I would not use such a questionnaire |

Q8. Do you think there is a need to further develop a quick and easy to use patient self-reported QoL tool that could be completed in the waiting room prior to each appointment? <ASK IF DQ7 = CODE 1>

Please select one answer

| ⭘ | Yes |
| --- | --- |
| ⭘ | No |

Q9. How frequently do/would you use this tool to monitor patients individual QoL? <ASK IF DQ7 = CODE 1 OR 2>

Please select one answer

| ⭘ | Every consultation |
| --- | --- |
| ⭘ | Every 6 months |
| ⭘ | Once a year |
| ⭘ | Less frequently than once a year |

Q10. Do you think a mobile phone app would be helpful for patients to record this information on the impact their ITP has on their QoL? <ASK ALL>

Please select one answer

| ⭘ | Yes |
| --- | --- |
| ⭘ | No |

Q11. What method of administration would ~~you~~ be most helpful for patients to record this information on the impact their ITP has on their QoL? <ASK ALL>

| ⭘ | Mobile phone application |
| --- | --- |
| ⭘ | Pen and paper questionnaire |
| ⭘ | Both would be preferable |

END OF SECTION

SECTION E: TREATMENT PATTERNS

Q1a. Please estimate in what proportion of your patients you choose to **observe** instead of recommending drug treatment following their first diagnosis of ITP (ITP>6 months duration)? <ASK ALL>

Please enter a percentage below

| _________ % of patients at diagnosis |
| --- |

Q1b. Among your previously treated patients beyond 12 months of diagnosis (or recurrent ITP), please estimate in what proportion of your patients you choose to observe instead of recommending drug treatment? <ASK ALL>

Please enter a percentage below

| _________ % of patients beyond 12 months (or recurrent) |
| --- |

Q1c. What are the 3 most common factors influencing your decision to observe some patients instead of recommending drug treatment following their initial diagnosis? <ASK IF EQ1a>0>

| 🞎 | Platelet levels not low enough to consider treatment |
| --- | --- |
| 🞎 | Patient bleeding symptoms were not severe enough to consider treatment |
| 🞎 | Patient was asymptomatic |
| 🞎 | Patient has another condition that would be affected by ITP drug treatment |
| 🞎 | Patient is receiving another treatment that would interact with the ITP drug treatment |
| 🞎 | Patient refuses to be treated |
| 🞎 | Other |

Q2. Which of the following treatments have you prescribed to your patients in the following groups to help manage their ITP?

1. Newly diagnosed ITP (prior to first relapse) <ASK IF S2d >0>
2. Persistent/Chronic/Recurrent <ASK ALL>

|  |  | a  Newly diagnosed | B  Persistent/ Chronic/ recurrent |
| --- | --- | --- | --- |
| 1 | Androgens (such as danazol) | 🞎 | 🞎 |
| 2 | Anti-CD20 (such as rituximab, veltuzumab or ofatumumab) | 🞎 | 🞎 |
| 3 | Anti-fibrinolytic (such as tranexamic acid or aminocaproic acid) | 🞎 | 🞎 |
| 4 | Other immunosuppressants (such as azathioprine,cyclophosphamide or mofetil) | 🞎 | 🞎 |
| 5 | Cyclosporine (such as cyclosporine A or cytoxin) |  |  |
| 6 | Intravenous Immunoglobulins (IVIg) | 🞎 | 🞎 |
| 7 | Platelet transfusion | 🞎 | 🞎 |
| 8 | Rho(D) immune globulin (Anti-D) | 🞎 | 🞎 |
| 9 | Corticosteroids / steroids (such as prednisolone, methylprednisolone or dexamethasone) | 🞎 | 🞎 |
| 10 | Thrombopoietin receptor agonists (such as romiplostim or eltrombopag) | 🞎 | 🞎 |
| 11 | Other | 🞎 | 🞎 |
| 12 | No therapy (watch and wait) | 🞎 | 🞎 |

Q3. In your experience, what proportion of the following patient groups would receive a splenectomy? <ASK ALL>

1. Newly diagnosed ITP (prior to first relapse) <ASK IF S2d >0>
2. Persistent/Chronic/Recurrent <ASK ALL>

Please enter a percentage below

| a) Newly diagnosed ITP (prior to first relapse | b) Persistent/Chronic/Recurrent |
| --- | --- |
| __________ % of patients | __________ % of patients |

Q4. Which treatments are you most likely to prescribe for a patient as a consequence of their first relapse, second relapse and third relapse? <ASK ALL>

**Please select up to a maximum of 5 treatments for each column, where 1 is the most preferred.**

|  |  | First relapse | Second relapse | Third relapse |
| --- | --- | --- | --- | --- |
| 1 | Androgens (such as danazol) | 🞎 | 🞎 | 🞎 |
| 2 | Anti-CD20 (such as rituximab, veltuzumab or ofatumumab) | 🞎 | 🞎 | 🞎 |
| 3 | Anti-fibrinolytic (such as tranexamic acid or aminocaproic acid) | 🞎 | 🞎 | 🞎 |
| 4 | Other immunosuppressants (such as azathioprine, cyclophosphamide or mofetil) | 🞎 | 🞎 | 🞎 |
| 5 | Cyclosporine (such as cyclosporine A or cytoxin) | 🞎 | 🞎 | 🞎 |
| 6 | Intravenous Immunoglobulins (IVIg) | 🞎 | 🞎 | 🞎 |
| 7 | Platelet transfusion | 🞎 | 🞎 | 🞎 |
| 8 | Rho(D) immune globulin (Anti-D) | 🞎 | 🞎 | 🞎 |
| 9 | Corticosteroids / steroids (such as prednisolone, methylprednisolone or dexamethasone) | 🞎 | 🞎 | 🞎 |
| 10 | Thrombopoietin receptor agonists (such as romiplostim or eltrombopag) | 🞎 | 🞎 | 🞎 |
| 11 | Other | 🞎 | 🞎 | 🞎 |
| 12 | No therapy (watch and wait) | 🞎 | 🞎 | 🞎 |

Q5. Typically, how frequently do you check the platelet counts for the following patient groups? <ASK ALL>

1. Newly diagnosed ITP (prior to first relapse) <ASK IF S2d >0>
2. Persistent/Chronic/Recurrent <ASK ALL>

| Every _________ | ⭘ Weeks | ⭘ Months | ⭘ Years | ⭘ Don’t know |
| --- | --- | --- | --- | --- |

Q6. Please select the 5 treatments you are most likely to prescribe to the following patient groups? <ASK ALL>

**Please select up to a maximum of 5 treatments for each column**

|  |  | Platelet level (x10^3^/µL<US Only>)(x10^9^/L) | | | | |
| --- | --- | --- | --- | --- | --- | --- |
|  |  | <10 | 11-20 | 21-30 | 31-50 | 51-100 |
| 1 | Androgens (such as danazol) |  |  |  |  |  |
| 2 | Anti-CD20 (such as rituximab, veltuzumab or ofatumumab) |  |  |  |  |  |
| 3 | Anti-fibrinolytic (such as tranexamic acid or aminocaproic acid) |  |  |  |  |  |
| 4 | Other immunosuppressants (such as azathioprine, cyclophosphamide or mofetil) |  |  |  |  |  |
| 5 | Cyclosporine (such as cyclosporine A or cytoxin) |  |  |  |  |  |
| 6 | Intravenous Immunoglobulins (IVIg) |  |  |  |  |  |
| 7 | Platelet transfusion |  |  |  |  |  |
| 8 | Rho(D) immune globulin (Anti-D) |  |  |  |  |  |
| 9 | Corticosteroids / steroids (such as prednisolone, methylprednisolone or dexamethasone) |  |  |  |  |  |
| 10 | Thrombopoietin receptor agonists (such as romiplostim or eltrombopag) |  |  |  |  |  |
| 11 | Other |  |  |  |  |  |
| 12 | No therapy (watch and wait) |  |  |  |  |  |

Q7a. Of the statements below, what is more important to you when making decisions for your ITP patients? <ASK ALL>

**Please indicate on a scale of 1 to 100, where 1 = not important at all and 100 = of the highest importance**

| a | Prevention of immunosuppression | 100 point sliding scale |
| --- | --- | --- |
| b | Reduction of bleeding risk | 100 point sliding scale |
| c | Quick recovery but with a risk of recovery being temporary | 100 point sliding scale |
| d | Slower recovery from relapse, but potentially longer term | 100 point sliding scale |
| e | Keeping side effect to a minimum | 100 point sliding scale |
| f | Ability to offer sustained remission or cure of ITP | 100 point sliding scale |

Q7b. Please indicate your level of agreement with the following statement. <ASK ALL>

Please rate each statement below, where 1 (not at all); to 7 (a great deal)

|  | 1 | 2 | 3 | 4 | 5 | 6 | 7 |
| --- | --- | --- | --- | --- | --- | --- | --- |
| When discussing treatment options with my patients I explain the relative chances of sustained remission with each treatment class~~’~~ | ⭘ | ⭘ | ⭘ | ⭘ | ⭘ | ⭘ | ⭘ |

Q7c. In your opinion what proportion of patients experience a lasting remission following successful treatment with (or discontinuation of) the following: <ASK ALL>

| Splenectomy | ________ % |
| --- | --- |
| Anti-CD20s | ________ % |
| Thrombopoietin receptor agonists | ________ % |
| Corticosteroids/steroids | ________ % |
| Other immunosuppressants | ________ % |
| Intravenous Immunoglobulins |  |
| Other | ________ % |

Q7d.What is your preferred class of treatment for ITP if sustained remission is your goal? <ASK ALL>

| Splenectomy | ⭘ |
| --- | --- |
| Anti-CD20s | ⭘ |
| Thrombopoietin receptor agonists | ⭘ |
| Corticosteroids/steroids | ⭘ |
| Other immunosuppressants | ⭘ |
| Intravenous Immunoglobulins | ⭘ |
| Other | ⭘ |

Q8. What are the most commonly encountered side effects for the following treatments? <ASK ALL>

**Select 3 of the most commonly reported side effects from the list below.**

1. Anti-CD20

| 1 | 🞎 | Abnormal hunger | |
| --- | --- | --- | --- |
| 2 | 🞎 | Acne | |
| 3 | 🞎 | Anger and/or irritability | |
| 4 | 🞎 | Anxiety and/or nervousness | |
| 5 | 🞎 | Back aches and pains | |
| 6 | 🞎 | Body aches and pains (joint stiffness, muscle cramps) | |
| 7 | 🞎 | Bruising around injection site | |
| 8 | 🞎 | Changes in face shape, bloating, swelling | |
| 9 | 🞎 | Change in taste in my mouth | |
| 10 | 🞎 | Chills | |
| 11 | 🞎 | Cough | |
| 12 | 🞎 | Depression | |
| 13 | 🞎 | Diarrhea | |
| 14 | 🞎 | Difficulty sleeping | |
| 15 | 🞎 | Dizziness | |
| 16 | 🞎 | Fatigue | |
| 17 | 🞎 | Fever | |
| 18 | 🞎 | Generalized weakness | |
| 19 | 🞎 | Hair loss | |
| 20 | 🞎 | Headaches | |
| 21 | 🞎 | Heartburn | |
| 22 | 🞎 | Heavy menstrual bleeding | |
| 23 | 🞎 | High blood pressure | |
| 24 | 🞎 | Hirsutism (increased hair on face or body) | |
| 25 | 🞎 | Hot flushes and/or sweating | |
| 26 | 🞎 | Impaired wound healing | |
| 27 | 🞎 | Increased infections, either frequency of or length of time they last | |
| 28 | 🞎 | Increased thirst or urination | |
| 29 | 🞎 | Infusion reactions during the infusion such as fever, chills, abdominal pain | |
| 30 | 🞎 | Insomnia, restlessness and/or trouble sleeping | |
| 31 | 🞎 | Muscle weakness | |
| 32 | 🞎 | Nasopharyngitis (inflammation of the nasal cavities and/or throat) | |
| 33 | 🞎 | Nausea, upset stomach, vomiting, | |
| 34 | 🞎 | Night sweats | |
| 35 | 🞎 | Osteoporosis (reduction in bone density) | |
| 36 | 🞎 | Kidney disease | |
| 37 | 🞎 | Respiratory symptoms (e.g. breathlessness) | |
| 38 | 🞎 | Skin rash | |
| 39 | 🞎 | Skin thinning | |
| 40 | 🞎 | Stretch marks | |
| 41 | 🞎 | Swelling of the hands or feet | |
| 42 | 🞎 | Thrombosis (clotting too much) | |
| 43 | 🞎 | Tingling of the hands or feet | |
| 44 | 🞎 | Trouble with blood glucose levels, diabetes | |
| 45 | 🞎 | Vision problems (light sensitivity/decreased ability to see or read/cataracts/increased eye pressure (glaucoma) | |
| 46 | 🞎 | Weight gain / increased appetite |  |
| 47 | 🞎 | Weight loss |  |
| 48 | ⭘ | None |  |

1. i) Corticosteroids

| 1 | 🞎 | Abnormal hunger | |
| --- | --- | --- | --- |
| 2 | 🞎 | Acne | |
| 3 | 🞎 | Anger and/or irritability | |
| 4 | 🞎 | Anxiety and/or nervousness | |
| 5 | 🞎 | Back aches and pains | |
| 6 | 🞎 | Body aches and pains (joint stiffness, muscle cramps) | |
| 7 | 🞎 | Bruising around injection site | |
| 8 | 🞎 | Changes in face shape, bloating, swelling | |
| 9 | 🞎 | Change in taste in my mouth | |
| 10 | 🞎 | Chills | |
| 11 | 🞎 | Cough | |
| 12 | 🞎 | Depression | |
| 13 | 🞎 | Diarrhea | |
| 14 | 🞎 | Difficulty sleeping | |
| 15 | 🞎 | Dizziness | |
| 16 | 🞎 | Fatigue | |
| 17 | 🞎 | Fever | |
| 18 | 🞎 | Generalized weakness | |
| 19 | 🞎 | Hair loss | |
| 20 | 🞎 | Headaches | |
| 21 | 🞎 | Heartburn | |
| 22 | 🞎 | Heavy menstrual bleeding | |
| 23 | 🞎 | High blood pressure | |
| 24 | 🞎 | Hirsutism (increased hair on face or body) | |
| 25 | 🞎 | Hot flushes and/or sweating | |
| 26 | 🞎 | Impaired wound healing | |
| 27 | 🞎 | Increased infections, either frequency of or length of time they last | |
| 28 | 🞎 | Increased thirst or urination | |
| 29 | 🞎 | Infusion reactions during the infusion such as fever, chills, abdominal pain | |
| 30 | 🞎 | Insomnia, restlessness and/or trouble sleeping | |
| 31 | 🞎 | Muscle weakness | |
| 32 | 🞎 | Nasopharyngitis (inflammation of the nasal cavities and/or throat) | |
| 33 | 🞎 | Nausea, upset stomach, vomiting, | |
| 34 | 🞎 | Night sweats | |
| 35 | 🞎 | Osteoporosis (reduction in bone density) | |
| 36 | 🞎 | Kidney disease | |
| 37 | 🞎 | Respiratory symptoms (e.g. breathlessness) | |
| 38 | 🞎 | Skin rash | |
| 39 | 🞎 | Skin thinning | |
| 40 | 🞎 | Stretch marks | |
| 41 | 🞎 | Swelling of the hands or feet | |
| 42 | 🞎 | Thrombosis (clotting too much) | |
| 43 | 🞎 | Tingling of the hands or feet | |
| 44 | 🞎 | Trouble with blood glucose levels, diabetes | |
| 45 | 🞎 | Vision problems (light sensitivity/decreased ability to see or read/cataracts/increased eye pressure (glaucoma) | |
| 46 | 🞎 | Weight gain / increased appetite |  |
| 47 | 🞎 | Weight loss |  |
| 48 | ⭘ | None |  |

1. ii) Please state which side-effects of corticosteroids increase, remain at the same level or subside with further use. Only consider your patients where corticosteroids are recycled with multiple courses over several years. <SC PER ROW. ONLY SHOW SIDE EFFECTS SELECTED AT EQ4bii. IF ‘NOT APPLICABLE SELECTED DO NOT ALLOW ANY OTHER OPTIONS TO BE SELECTED>

|  |  | Increase | Remain at the same level | Subside |
| --- | --- | --- | --- | --- |
| 1 | Abnormal hunger | ⭘ | ⭘ | ⭘ |
| 2 | Acne | ⭘ | ⭘ | ⭘ |
| 3 | Anger and/or irritability | ⭘ | ⭘ | ⭘ |
| 4 | Anxiety and/or nervousness | ⭘ | ⭘ | ⭘ |
| 5 | Back aches and pains | ⭘ | ⭘ | ⭘ |
| 6 | Body aches and pains (joint stiffness, muscle cramps) | ⭘ | ⭘ | ⭘ |
| 7 | Bruising around injection site | ⭘ | ⭘ | ⭘ |
| 8 | Changes in face shape, bloating, swelling | ⭘ | ⭘ | ⭘ |
| 9 | Change in taste in my mouth | ⭘ | ⭘ | ⭘ |
| 10 | Chills | ⭘ | ⭘ | ⭘ |
| 11 | Cough | ⭘ | ⭘ | ⭘ |
| 12 | Depression | ⭘ | ⭘ | ⭘ |
| 13 | Diarrhea | ⭘ | ⭘ | ⭘ |
| 14 | Difficulty sleeping | ⭘ | ⭘ | ⭘ |
| 15 | Dizziness | ⭘ | ⭘ | ⭘ |
| 16 | Fatigue | ⭘ | ⭘ | ⭘ |
| 17 | Fever | ⭘ | ⭘ | ⭘ |
| 18 | Generalized weakness | ⭘ | ⭘ | ⭘ |
| 19 | Hair loss | ⭘ | ⭘ | ⭘ |
| 20 | Headaches | ⭘ | ⭘ | ⭘ |
| 21 | Heartburn | ⭘ | ⭘ | ⭘ |
| 22 | Heavy menstrual bleeding | ⭘ | ⭘ | ⭘ |
| 23 | High blood pressure | ⭘ | ⭘ | ⭘ |
| 24 | Hirsutism (increased hair on face or body) | ⭘ | ⭘ | ⭘ |
| 25 | Hot flushes and/or sweating | ⭘ | ⭘ | ⭘ |
| 26 | Impaired wound healing | ⭘ | ⭘ | ⭘ |
| 27 | Increased infections, either frequency of or length of time they last | ⭘ | ⭘ | ⭘ |
| 28 | Increased thirst or urination | ⭘ | ⭘ | ⭘ |
| 29 | Infusion reactions during the infusion such as fever, chills, abdominal pain | ⭘ | ⭘ | ⭘ |
| 30 | Insomnia, restlessness and/or trouble sleeping | ⭘ | ⭘ | ⭘ |
| 31 | Muscle weakness | ⭘ | ⭘ | ⭘ |
| 32 | Nasopharyngitis (inflammation of the nasal cavities and/or throat) | ⭘ | ⭘ | ⭘ |
| 33 | Nausea, upset stomach, vomiting, | ⭘ | ⭘ | ⭘ |
| 34 | Night sweats | ⭘ | ⭘ | ⭘ |
| 35 | Osteoporosis (reduction in bone density) | ⭘ | ⭘ | ⭘ |
| 36 | Kidney disease | ⭘ | ⭘ | ⭘ |
| 37 | Respiratory symptoms (e.g. breathlessness) | ⭘ | ⭘ | ⭘ |
| 38 | Skin rash | ⭘ | ⭘ | ⭘ |
| 39 | Skin thinning | ⭘ | ⭘ | ⭘ |
| 40 | Stretch marks | ⭘ | ⭘ | ⭘ |
| 41 | Swelling of the hands or feet | ⭘ | ⭘ | ⭘ |
| 42 | Thrombosis (clotting too much) | ⭘ | ⭘ | ⭘ |
| 43 | Tingling of the hands or feet | ⭘ | ⭘ | ⭘ |
| 44 | Trouble with blood glucose levels, diabetes | ⭘ | ⭘ | ⭘ |
| 45 | Vision problems (light sensitivity/decreased ability to see or read/cataracts/increased eye pressure (glaucoma) | ⭘ | ⭘ | ⭘ |
| 46 | Weight gain / increased appetite | ⭘ | ⭘ | ⭘ |
| 47 | Weight loss | ⭘ | ⭘ | ⭘ |
| 48 | None | ⭘ | ⭘ | ⭘ |

1. Splenectomy

| 1 | 🞎 | Abnormal hunger | |
| --- | --- | --- | --- |
| 2 | 🞎 | Acne | |
| 3 | 🞎 | Anger and/or irritability | |
| 4 | 🞎 | Anxiety and/or nervousness | |
| 5 | 🞎 | Back aches and pains | |
| 6 | 🞎 | Body aches and pains (joint stiffness, muscle cramps) | |
| 7 | 🞎 | Bruising around injection site | |
| 8 | 🞎 | Changes in face shape, bloating, swelling | |
| 9 | 🞎 | Change in taste in my mouth | |
| 10 | 🞎 | Chills | |
| 11 | 🞎 | Cough | |
| 12 | 🞎 | Depression | |
| 13 | 🞎 | Diarrhea | |
| 14 | 🞎 | Difficulty sleeping | |
| 15 | 🞎 | Dizziness | |
| 16 | 🞎 | Fatigue | |
| 17 | 🞎 | Fever | |
| 18 | 🞎 | Generalized weakness | |
| 19 | 🞎 | Hair loss | |
| 20 | 🞎 | Headaches | |
| 21 | 🞎 | Heartburn | |
| 22 | 🞎 | Heavy menstrual bleeding | |
| 23 | 🞎 | High blood pressure | |
| 24 | 🞎 | Hirsutism (increased hair on face or body) | |
| 25 | 🞎 | Hot flushes and/or sweating | |
| 26 | 🞎 | Impaired wound healing | |
| 27 | 🞎 | Increased infections, either frequency of or length of time they last | |
| 28 | 🞎 | Increased thirst or urination | |
| 29 | 🞎 | Infusion reactions during the infusion such as fever, chills, abdominal pain | |
| 30 | 🞎 | Insomnia, restlessness and/or trouble sleeping | |
| 31 | 🞎 | Muscle weakness | |
| 32 | 🞎 | Nasopharyngitis (inflammation of the nasal cavities and/or throat) | |
| 33 | 🞎 | Nausea, upset stomach, vomiting, | |
| 34 | 🞎 | Night sweats | |
| 35 | 🞎 | Osteoporosis (reduction in bone density) | |
| 36 | 🞎 | Kidney disease | |
| 37 | 🞎 | Respiratory symptoms (e.g. breathlessness) | |
| 38 | 🞎 | Skin rash | |
| 39 | 🞎 | Skin thinning | |
| 40 | 🞎 | Stretch marks | |
| 41 | 🞎 | Swelling of the hands or feet | |
| 42 | 🞎 | Thrombosis (clotting too much) | |
| 43 | 🞎 | Tingling of the hands or feet | |
| 44 | 🞎 | Trouble with blood glucose levels, diabetes | |
| 45 | 🞎 | Vision problems (light sensitivity/decreased ability to see or read/cataracts/increased eye pressure (glaucoma) | |
| 46 | 🞎 | Weight gain / increased appetite |  |
| 47 | 🞎 | Weight loss |  |
| 48 | ⭘ | None |  |

1. Thrombopoietin receptor agonists

| 1 | 🞎 | Abnormal hunger | |
| --- | --- | --- | --- |
| 2 | 🞎 | Acne | |
| 3 | 🞎 | Anger and/or irritability | |
| 4 | 🞎 | Anxiety and/or nervousness | |
| 5 | 🞎 | Back aches and pains | |
| 6 | 🞎 | Body aches and pains (joint stiffness, muscle cramps) | |
| 7 | 🞎 | Bruising around injection site | |
| 8 | 🞎 | Changes in face shape, bloating, swelling | |
| 9 | 🞎 | Change in taste in my mouth | |
| 10 | 🞎 | Chills | |
| 11 | 🞎 | Cough | |
| 12 | 🞎 | Depression | |
| 13 | 🞎 | Diarrhea | |
| 14 | 🞎 | Difficulty sleeping | |
| 15 | 🞎 | Dizziness | |
| 16 | 🞎 | Fatigue | |
| 17 | 🞎 | Fever | |
| 18 | 🞎 | Generalized weakness | |
| 19 | 🞎 | Hair loss | |
| 20 | 🞎 | Headaches | |
| 21 | 🞎 | Heartburn | |
| 22 | 🞎 | Heavy menstrual bleeding | |
| 23 | 🞎 | High blood pressure | |
| 24 | 🞎 | Hirsutism (increased hair on face or body) | |
| 25 | 🞎 | Hot flushes and/or sweating | |
| 26 | 🞎 | Impaired wound healing | |
| 27 | 🞎 | Increased infections, either frequency of or length of time they last | |
| 28 | 🞎 | Increased thirst or urination | |
| 29 | 🞎 | Infusion reactions during the infusion such as fever, chills, abdominal pain | |
| 30 | 🞎 | Insomnia, restlessness and/or trouble sleeping | |
| 31 | 🞎 | Muscle weakness | |
| 32 | 🞎 | Nasopharyngitis (inflammation of the nasal cavities and/or throat) | |
| 33 | 🞎 | Nausea, upset stomach, vomiting, | |
| 34 | 🞎 | Night sweats | |
| 35 | 🞎 | Osteoporosis (reduction in bone density) | |
| 36 | 🞎 | Kidney disease | |
| 37 | 🞎 | Respiratory symptoms (e.g. breathlessness) | |
| 38 | 🞎 | Skin rash | |
| 39 | 🞎 | Skin thinning | |
| 40 | 🞎 | Stretch marks | |
| 41 | 🞎 | Swelling of the hands or feet | |
| 42 | 🞎 | Thrombosis (clotting too much) | |
| 43 | 🞎 | Tingling of the hands or feet | |
| 44 | 🞎 | Trouble with blood glucose levels, diabetes | |
| 45 | 🞎 | Vision problems (light sensitivity/decreased ability to see or read/cataracts/increased eye pressure (glaucoma) | |
| 46 | 🞎 | Weight gain / increased appetite |  |
| 47 | 🞎 | Weight loss |  |
| 48 | ⭘ | None |  |

1. Intravenous Immunoglobins

| 1 | 🞎 | Abnormal hunger | |
| --- | --- | --- | --- |
| 2 | 🞎 | Acne | |
| 3 | 🞎 | Anger and/or irritability | |
| 4 | 🞎 | Anxiety and/or nervousness | |
| 5 | 🞎 | Back aches and pains | |
| 6 | 🞎 | Body aches and pains (joint stiffness, muscle cramps) | |
| 7 | 🞎 | Bruising around injection site | |
| 8 | 🞎 | Changes in face shape, bloating, swelling | |
| 9 | 🞎 | Change in taste in my mouth | |
| 10 | 🞎 | Chills | |
| 11 | 🞎 | Cough | |
| 12 | 🞎 | Depression | |
| 13 | 🞎 | Diarrhea | |
| 14 | 🞎 | Difficulty sleeping | |
| 15 | 🞎 | Dizziness | |
| 16 | 🞎 | Fatigue | |
| 17 | 🞎 | Fever | |
| 18 | 🞎 | Generalized weakness | |
| 19 | 🞎 | Hair loss | |
| 20 | 🞎 | Headaches | |
| 21 | 🞎 | Heartburn | |
| 22 | 🞎 | Heavy menstrual bleeding | |
| 23 | 🞎 | High blood pressure | |
| 24 | 🞎 | Hirsutism (increased hair on face or body) | |
| 25 | 🞎 | Hot flushes and/or sweating | |
| 26 | 🞎 | Impaired wound healing | |
| 27 | 🞎 | Increased infections, either frequency of or length of time they last | |
| 28 | 🞎 | Increased thirst or urination | |
| 29 | 🞎 | Infusion reactions during the infusion such as fever, chills, abdominal pain | |
| 30 | 🞎 | Insomnia, restlessness and/or trouble sleeping | |
| 31 | 🞎 | Muscle weakness | |
| 32 | 🞎 | Nasopharyngitis (inflammation of the nasal cavities and/or throat) | |
| 33 | 🞎 | Nausea, upset stomach, vomiting, | |
| 34 | 🞎 | Night sweats | |
| 35 | 🞎 | Osteoporosis (reduction in bone density) | |
| 36 | 🞎 | Kidney disease | |
| 37 | 🞎 | Respiratory symptoms (e.g. breathlessness) | |
| 38 | 🞎 | Skin rash | |
| 39 | 🞎 | Skin thinning | |
| 40 | 🞎 | Stretch marks | |
| 41 | 🞎 | Swelling of the hands or feet | |
| 42 | 🞎 | Thrombosis (clotting too much) | |
| 43 | 🞎 | Tingling of the hands or feet | |
| 44 | 🞎 | Trouble with blood glucose levels, diabetes | |
| 45 | 🞎 | Vision problems (light sensitivity/decreased ability to see or read/cataracts/increased eye pressure (glaucoma) | |
| 46 | 🞎 | Weight gain / increased appetite |  |
| 47 | 🞎 | Weight loss |  |
| 48 | ⭘ | None |  |

Q9a. How satisfied are you with the treatment and therapy options available for ITP? <ASK ALL>

Please provide a rating below, where 1 (completely unsatisfied); to 7 (completely satisfied)

|  |  | 1 | 2 | 3 | 4 | 5 | 6 | 7 |
| --- | --- | --- | --- | --- | --- | --- | --- | --- |
|  | Satisfaction with current treatment options for ITP | ⭘ | ⭘ | ⭘ | ⭘ | ⭘ | ⭘ | ⭘ |

Q9b. Why are you not satisfied with the current treatment options for ITP?

Please select all that apply

| 1 | 🞎 | Limited treatment options |
| --- | --- | --- |
| 2 | 🞎 | Not able to reach the treatment goals I set for my patients with current treatment options |
| 3 | 🞎 | Do not improve disease symptoms |
| 4 | 🞎 | Unacceptable side effects |
| 5 | 🞎 | Do not improve quality of life for patients |
| 6 | 🞎 | Not able to provide deep/stable remission for patients |
| 7 | 🞎 | Lack of efficacy |
| 8 | 🞎 | Safety concerns around treatment options |
| 9 | 🞎 | Patient’s become refractory to treatment |
| 10 | 🞎 | Other |

Q9c. Why are you satisfied with the current treatment options for ITP?

Please select all that apply

| 1 | 🞎 | Wide selection of treatments |
| --- | --- | --- |
| 2 | 🞎 | Able to reach treatment goals I set for my patients with current treatment options |
| 3 | 🞎 | Improve disease symptoms |
| 4 | 🞎 | Few side effects from treatment |
| 5 | 🞎 | Improves quality of life for patients |
| 6 | 🞎 | Provides deep/stable remission for patients |
| 7 | 🞎 | Highly efficacious |
| 8 | 🞎 | Other |

Q10. Other than a cure for ITP, what are your 3 most important treatment goals for your patients? <ASK ALL>

Please assign rankings (1 to 3) with 1 being the most important

| 1 | 🞎 | Reduce fatigue |
| --- | --- | --- |
| 2 | 🞎 | Reduction in spontaneous bleeds |
| 3 | 🞎 | Healthy blood counts (i.e. stable platelet count) |
| 4 | 🞎 | Slow/delay progression of disease |
| 5 | 🞎 | Better quality of life |
| 6 | 🞎 | Symptom improvement |
| 7 | 🞎 | Lighter menstrual periods |
| 8 | 🞎 | Reduce general weakness |
| 9 | 🞎 | Prevention of blood clots |
| 10 | 🞎 | Convenience of treatment administration |
| 11 | 🞎 | Ease of storing/transporting product (IV vs. tablet) |

Q11. To what extent do you agree with the following: <ASK ALL>

Please rate each statement, where 1 (strongly disagree); to 7 (strongly agree)

|  |  | 1 | 2 | 3 | 4 | 5 | 6 | 7 |
| --- | --- | --- | --- | --- | --- | --- | --- | --- |
| 1 | My ITP patients understand the treatment goals I have set out | ⭘ | ⭘ | ⭘ | ⭘ | ⭘ | ⭘ | ⭘ |
| 2 | I discuss treatment goals with my ITP patients and we agree on these together | ⭘ | ⭘ | ⭘ | ⭘ | ⭘ | ⭘ | ⭘ |
| 3 | My main aim of ITP treatment is to limit immunosuppressive side effects of treatment | ⭘ | ⭘ | ⭘ | ⭘ | ⭘ | ⭘ | ⭘ |

Q12. In what circumstances would you change drug therapy for your patient? <ASK ALL>

Please select all that apply

| 1 | 🞎 | Lack of efficacy |
| --- | --- | --- |
| 2 | 🞎 | Disease progression |
| 3 | 🞎 | Side effects |
| 4 | 🞎 | Change in blood counts |
| 5 | 🞎 | Patient preference |
| 6 | 🞎 | Cost/coverage |
| 7 | 🞎 | Change of symptoms |
| 8 | 🞎 | Other |

Q13. Please state the order of preference for the following treatment options for your ITP patients?

<ASK ALL>

Please add 1 for the most preferred option, 2 for the second and 3 for the third. <PROGRAMME RANKING OF 1-3 ONLY>

|  | Newly diagnosed (prior to relapse) | Persistent/chronic/recurrent |
| --- | --- | --- |
| Oral treatment options | ---- | ---- |
| Subcutaneous treatment options | ---- | ---- |
| Intravenous treatment options | ---- | ---- |

END OF SECTION

SECTION F: PATIENT AND PHYSICIAN RELATIONSHIP

Q1. Thinking about your ITP patients, overall how satisfied do you feel your patients are with the following aspects of their care and management of their ITP? <ASK ALL>

Please rate each statement below, where 1 (completely dissatisfied); to 7 (completely satisfied)

|  |  | 1 | 2 | 3 | 4 | 5 | 6 | 7 |
| --- | --- | --- | --- | --- | --- | --- | --- | --- |
| 1 | Treatment decisions that have been made | ⭘ | ⭘ | ⭘ | ⭘ | ⭘ | ⭘ | ⭘ |
| 2 | Treatment goals that have been set out | ⭘ | ⭘ | ⭘ | ⭘ | ⭘ | ⭘ | ⭘ |
| 3 | Your management of their condition, i.e. symptoms | ⭘ | ⭘ | ⭘ | ⭘ | ⭘ | ⭘ | ⭘ |
| 4 | Your communications about their disease and treatment | ⭘ | ⭘ | ⭘ | ⭘ | ⭘ | ⭘ | ⭘ |
| 5 | Ease and convenience of prescribed medication | ⭘ | ⭘ | ⭘ | ⭘ | ⭘ | ⭘ | ⭘ |

Q2. Overall, based on your experience with your consulting ITP patients, how much do you agree with the following statements? <ASK ALL>

Please rate each statement below, where 1 (strongly disagree); to 7 (strongly agree).

|  |  | 1 | 2 | 3 | 4 | 5 | 6 | 7 |
| --- | --- | --- | --- | --- | --- | --- | --- | --- |
| 1 | My ITP patients feel that I am genuinely concerned about helping them | ⭘ | ⭘ | ⭘ | ⭘ | ⭘ | ⭘ | ⭘ |
| 2 | My ITP patients feel that I really listen to concerns and address questions | ⭘ | ⭘ | ⭘ | ⭘ | ⭘ | ⭘ | ⭘ |
| 3 | I am aware of my patients’ concerns around immunosuppression | ⭘ | ⭘ | ⭘ | ⭘ | ⭘ | ⭘ | ⭘ |
| 4 | I keep my patients informed about new treatment options | ⭘ | ⭘ | ⭘ | ⭘ | ⭘ | ⭘ | ⭘ |
| 5 | I create treatment plans for therapy that I discuss with my respective patient | ⭘ | ⭘ | ⭘ | ⭘ | ⭘ | ⭘ | ⭘ |
| 6 | I ask my ITP patients about their symptoms at every appointment | ⭘ | ⭘ | ⭘ | ⭘ | ⭘ | ⭘ | ⭘ |
| 7 | I understand how much ITP condition affects my patients’ lives | ⭘ | ⭘ | ⭘ | ⭘ | ⭘ | ⭘ | ⭘ |
| 8 | My patients feel comfortable discussing their symptoms with me | ⭘ | ⭘ | ⭘ | ⭘ | ⭘ | ⭘ | ⭘ |
| 9 | There is always time during the consultation to discuss all of the symptoms a patient is experiencing | ⭘ | ⭘ | ⭘ | ⭘ | ⭘ | ⭘ | ⭘ |
| 10 | I explain test results to my ITP patients and how they could be impacting their symptoms | ⭘ | ⭘ | ⭘ | ⭘ | ⭘ | ⭘ | ⭘ |
| 11 | My patients are able to accurately describe their symptoms to me | ⭘ | ⭘ | ⭘ | ⭘ | ⭘ | ⭘ | ⭘ |
| 12 | I am confident that I am thoroughly assessing and treating my patients | ⭘ | ⭘ | ⭘ | ⭘ | ⭘ | ⭘ | ⭘ |
| 13 | I have similar goals for treatment as my respective patients | ⭘ | ⭘ | ⭘ | ⭘ | ⭘ | ⭘ | ⭘ |
| 14 | I explain the possible long-term complications of a splenectomy to my patients before they undergo the procedure | ⭘ | ⭘ | ⭘ | ⭘ | ⭘ | ⭘ | ⭘ |
| 15 | I explain the potential drug side effects to a patient before giving a treatment | ⭘ | ⭘ | ⭘ | ⭘ | ⭘ | ⭘ | ⭘ |
| 16 | I am aware of my patients concerns about their treatments | ⭘ | ⭘ | ⭘ | ⭘ | ⭘ | ⭘ | ⭘ |
| 17 | I am confident that I am able to keep up-to-date with therapy options in ITP | ⭘ | ⭘ | ⭘ | ⭘ | ⭘ | ⭘ | ⭘ |

Q3. How easy is it for patient to access clear and relevant information about their ITP condition, outside of your practice/clinic? <ASK ALL>

Please select one answer

| ⭘ | Very easily accessible |
| --- | --- |
| ⭘ | Somewhat accessible |
| ⭘ | Accessible with some difficulty |
| ⭘ | Very difficult to find any information outside of the practice/clinic to inform patients |
| ⭘ | Don’t know |

Q4. Which of the following, if any, do you provide your patients with? <ASK ALL>

Please select one answer

| ⭘ | I provide patients with an ITP information leaflet and explain the content |
| --- | --- |
| ⭘ | I provide patients with an ITP information leaflet, but I do not review the content |
| ⭘ | I show an ITP specific website during the consultation and explain the content to the patient |
| ⭘ | I direct patients to an ITP website(s) with more information on ITP |
| ⭘ | I direct patients to research Patient Support Groups in ITP so that they can find out more about their disease |
| ⭘ | The nurse usually provides ITP specific information to patients, not me. |
| ⭘ | Information about enrolling in clinical trials |
| ⭘ | None of the above |

END OF SURVEY
